# Temporal and spatial order of photoreceptor and glia projections into optic lobe in *Drosophila*

**DOI:** 10.1038/s41598-018-30415-8

**Published:** 2018-08-23

**Authors:** Yen-Ching Chang, Chia-Kang Tsao, Y. Henry Sun

**Affiliations:** 1 Institute of Genomic Sciences, National Yang Ming University, Taipei, Taiwan; 2 Institute of Molecular Biology, Academia Sinica, Taipei, Taiwan

## Abstract

Photoreceptor (PR) axons project from the retina to the optic lobe in brain and form a precise retinotopic map in the *Drosophila* visual system. Yet the role of retinal basal glia in the retinotopic map formation is not previously known. We examined the formation of the retinotopic map by marking single PR pairs and following their axonal projections. In addition to confirming previous studies that the spatial information is preserved from the retina to the optic stalk and then to the optic lamina, we found that the young PR R3/4 axons transiently overshoot and then retract to their final destination, the lamina plexus. We then examined the process of wrapping glia (WG) membrane extension in the eye disc and showed that the WG membrane extensions also follow the retinotopic map. We show that the WG is important for the proper spatial distribution of PR axons in the optic stalk and lamina, suggesting an active role of wrapping glia in the retinotopic map formation.

## Introduction

The visual system of both vertebrates and invertebrates consists of light-sensing photoreceptor (PR) neurons in the retina connected to the inner layers of neurons to form a precise retinotopic map for visual information processing^1–3^. In the vertebrate retina, the PR axons project inward to form synapses with bipolar cells at the outer plexiform layer. The bipolar cells then project inward to make synaptic connections with the retinal ganglion cells (RGC) at the inner plexiform layer. Within each layer, the neurons are connected by interneurons for information integration. The RGCs axons then exit the retina and make connections with the optic tectum or lateral geniculate nucleus (LGN) in the brain in a spatially precise one-to-one retinotopic map. The retinotopic map preserves the spatial information detected by the PR and transmit into the brain for further information processing^1–3^. The formation of the retinotopic map is dependent on gradients of guidance molecules and on activity-dependent interactions among axons^1–4^.

**Figure 1:**
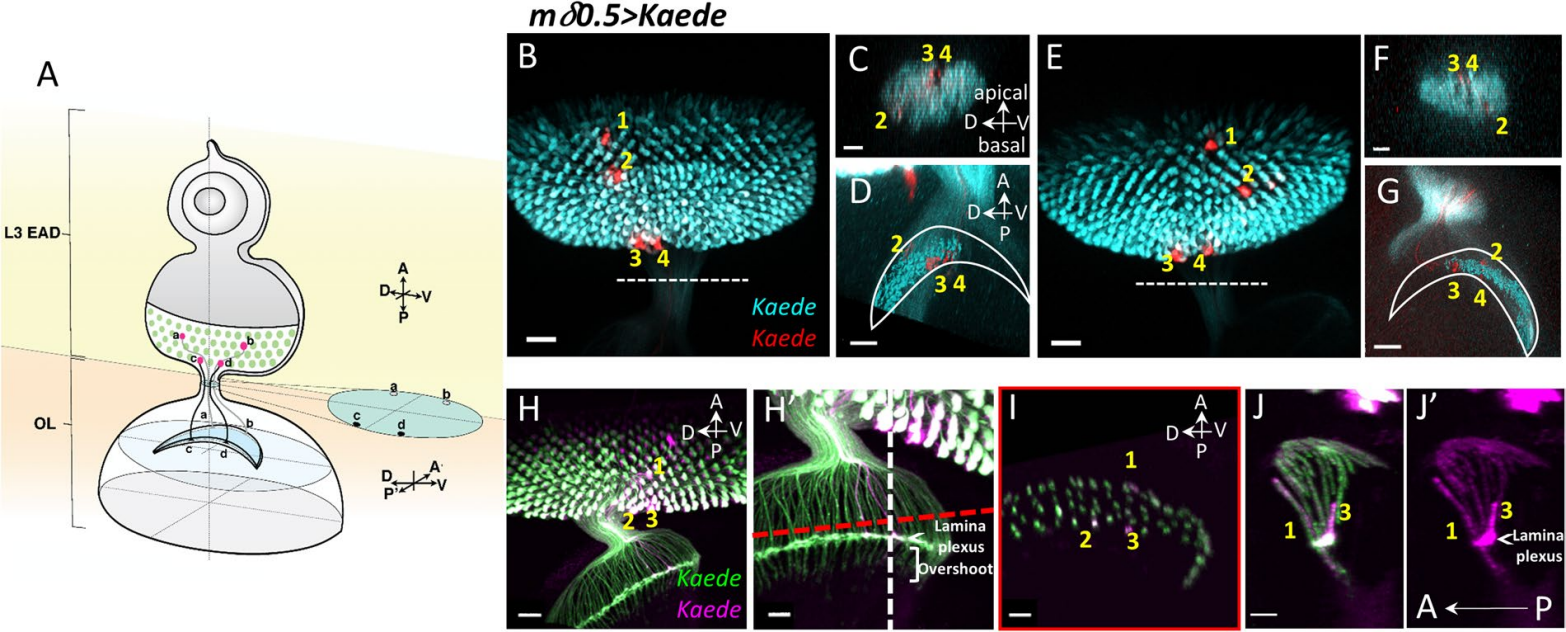
Retinotopic mapping of PR axonal projections in OS and optic lamina. *Kaede* expression in R3/4 is driven by *mδ0.5-GAL4*. (**A**) Schematic drawing summarizes the axon projections of single R3/4 pairs from different A-P and D-V positions in the eye disc into the OS and lamina. Samples were either live discs mounted in low gelling agarose (**B**–**G**) or fixed discs mounted on slides (**H**–**J**) (N = 7 for live discs and N = 10 for fixed discs). At least 4 single R3/4 pairs were analyzed for each disc. All respected their relative A-P and D-V order. The fixed samples provided better resolution in lamina. Axon projections were traced through different Z-sections. (H-J) Two copies of *mδ0.5-GAL4* were used. (**B**–**D**), (**E**–**G**) and (**H**–**J**) are three disc samples. (**B**,**E**) Z-projections of XY plane are shown to indicate four photoactivated positions (numbered 1–4) in each disc. (**C**,**F**) In the OS, the older axons (#3 and #4) are found in the apical region. In both samples, the most anterior #1 axon has not reached OS and lamina. The anterior-dorsal #2 axon (**B**) projects to basal-dorsal position (**C**). The anterior-ventral #2 axon (**D**) projects to basal-ventral position (**E**). (**D**,**G**) The labeled axon projections (red) in the lamina were 3D-reconstructed by IMARIS and merged with all R3/4 axons (cyan). These axons maintain their relative D-V and A-P positions as in retina. (**H**) Z-projection shows the position of three photo-activated R3/4 pairs (#1–3) and their axon projections in the lamina, with a higher magnification view of the lamina in the same sample from a different angle (H’). The 3D reconstruction is viewed in two optical cross sections at the red dashed line (**I**) and white dashed line (**J**,J’) in H’. (**I**) The #1–3 axons maintained their relative D-V and A-P positions. (**J**,J’) In this lateral view of the lamina, the #3 (posterior, older) axon terminate at the lamina plexus (white arrowhead), and the #1 (anterior, young) axon overshoot the lamina plexus. Cyan (green Kaede)/red (red Kaede) presentation is for sample in agarose. Green (green Kaede)/magenta (red Kaede) presentation is for fixed samples. Scale bars are 20 μm for BEH and 10 μm for CDFGH’IJJ’.

The highly regular and repeated structure of the *Drosophila* visual system makes it an excellent experimental model to study mechanisms regulating the formation of the retinotopic map. The *Drosophila* compound eye consists of around 800 ommatidia, each with eight photoreceptors (R1-8) plus a number of accessory cells^5^. The PR axons project into the optic lobe in a spatially precise one-to-one retinotopic map, i.e. maintaining their relative anterioposterior (A-P) and dorsoventral (D-V) order (Fig. 1A). The spatial precision of the PR axonal projections into the optic lobe has been examined in detail in the adult brain of larger insects and of *Drosophila*^6–11^. The fly eye also provides a great opportunity to study the developmental progression of axonal projections and the formation of the retinotopic map (reviewed by^12^). The fly compound eye develops from the larval eye imaginal disc, which is connected to the brain via the optic stalk (OS). The eye disc is a single epithelial cell layer, covered with a single apical peripodial membrane that does not contribute to the adult eye. During the third instar larval stage, the eye disc begins to differentiate in a progressive wave moving from the posterior end to the anterior portion. Cells at the front of the wave transiently shorten and form a morphogenetic furrow (MF) along the D-V direction. As the D-V-oriented MF moves anteriorly, cells behind the MF begin to progressively differentiate into rows of ommatidial clusters consisting of photoreceptors^5,13^. The PRs differentiate in the sequence of R8 - R2/5 - R3/4 - R1/6 - R7^13^. The newly differentiated PRs extend axons basally and then posteriorly along the basal surface, go through the OS and into the optic lobe. The R1-6 axons terminate in the optic lamina and the R7 and R8 axons extend further and enter the medulla^14^. The R1-6 growth cones terminate between the rows of epithelial glia and marginal glia, forming the lamina plexus^15^. During the pupal stage, the R1-6 axons defasciculate to undergo extensive rearrangement and make synapses with lamina neurons. The R7 and R8 axons terminate at different layers in the medulla and form synapses with lamina neurons^16–18^. In this study, we will focus on the formation of the first layer of the retinotopic map, i.e. the projection of PR axons to the lamina during the larval stage.

The overall organization and design principle of the *Drosophila* retina/lamina/medulla and the vertebrate retina/inner and outer plexiform layers is very similar^19–21^. Therefore, the study of fly retinotopy can provide useful insight for the understanding of the development of the mammalian visual system. Earlier studies have examined the retinotopic projections of PR axons by electron microscopy (EM) analysis in larger insects^8–10^. The retinotopic map in *Drosophila* was examined by using the lipophilic dyes DiI and DiO to label the dorsal and ventral PR, respectively, the *omb-τlacZ* to mark the dorsal- and ventral-most PR axons, and the temporal difference in expression timing of anti-horseradish peroxidase (HRP) and monoclonal antibody 24B10 staining to distinguish the younger and older axons, and followed their axonal projections into OS and optic lobe^22,23^. The HRP is a neuronal marker^24^ that stains the core α1-3-fucosylated N-glycan on neuronal cell surface proteins^25,26^. The 24B10 stains specifically PR cell body and axon^27^ by recognizing the cell surface glycoprotein Chaoptin^28,29^. Because the HRP signal appears in differentiating PR about 9 hr. (at 25 °C) before the 24B10 signal, the most anterior (youngest) six or seven rows of ommatidia are labeled by anti-HRP but not by 24B10^22^. Thus HRP and 24B10 double staining can be used to distinguish the younger and older axons^11^. Marked PR clones were also analyzed^30^. The results showed that the PR axons follow the A-P (younger-older) and D-V order in their projection into the OS and optic lamina^22^. However, because, a group of axons were labelled in these experiments, the spatial resolution of PR axon development is limited. In this study, we used the photoconvertible fluorescent protein Kaede^31–33^ to explore this question. UV irradiation induces a peptide cleavage of Kaede proteins, which results in the rapid and irreversible conversion from green fluorescence to red fluorescence^32^. By expressing Kaede in specific PRs and selectively photo-activating PR at specific positions, we can follow PR axonal projections into OS and lamina with single axonal pair resolution.

The vertebrate retinal neurons are ensheathed by Müller glia and astrocytes (reviewed by^34,35^ but the role of these glia in the formation of retinotopic map has not been reported. Glia is involved in the PR axonal projection in *Drosophila*. In the optic lamina, glia is required for the proper termination of R1-6 axons in the lamina plexus^36–38^. In the eye disc, a group of glia, the retinal basal glia (RBG), migrates from the OS into the basal layer of the eye disc^39,40^. Their migration into the eye disc follows the PR differentiation and they always lag behind the anterior front of the differentiating PR^41^. PR axons can project toward ectopic RBGs in the eye disc^41^, suggesting that RBGs may provide some guidance cues for PR axons. But whether the RBGs are required for PR axon projection is controversial. Expressing the dominant-negative Ras in RBG blocked RBG migration into eye disc and prevented PR axons from entering the OS^40^. In contrast, knocking down of the αPS2 and βPS integrin in RBG also blocked RBG migration into the eye disc, but the PR axons project normally^42^.

The PR axons do not project into optic lobe as naked axons; they are enwrapped by glial membrane. The RBG consist of three major glia cell types, namely carpet glia (CG), surface glia (SG) and wrapping glia (WG) that are distinguished by morphological and molecular characteristics^41,43,44^. The WGs differentiate from SG^44,45^ in response to FGF signaling^46^ and extend membrane to wrap around the PR axons. This wrapping process is dependent on FGFR signaling and on the interaction of two cell surface proteins: Borderless (Bdl) expressed in WG and Turtle (Tutl) expressed in PR^47,48^. However, whether the glial wrapping of PR axons follow the same retinotopic principle and whether the glial wrapping plays any role in PR axonal projection have not been studied. In this study, we examined the temporal and spatial progression of WG membrane wrapping of PR axons and addressed whether the wrapping affects PR axonal projection. Our results demonstrated axonal ensheathment by wrapping glia is critical for the retinotopic map formation.

## Results

### Retinotopic mapping of PR axonal projections in OS and optic lamina

**Figure 2:**
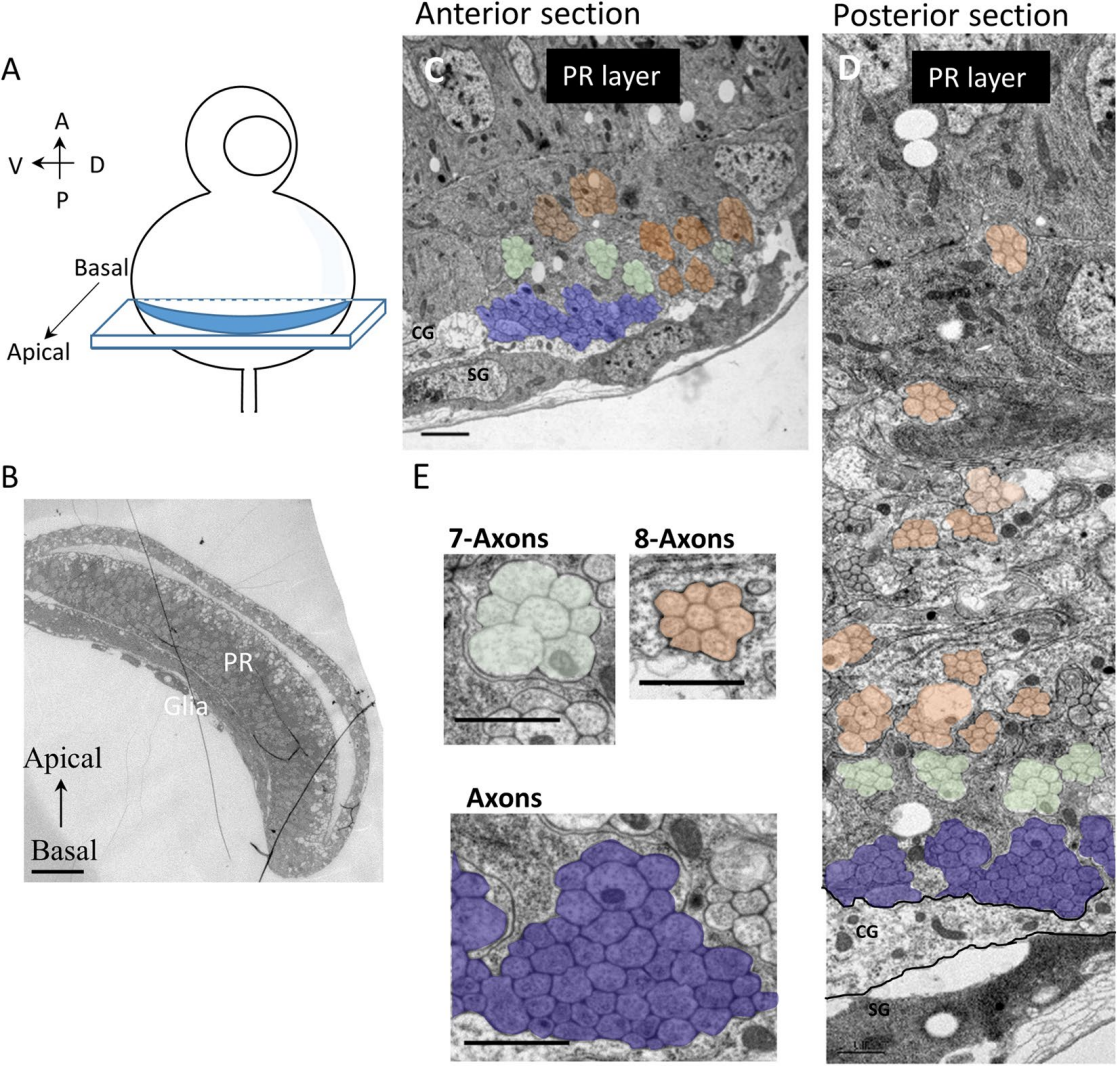
Young-old PR axons follow basal-apical order in the basal layer of eye disc. (**A**) Schematic diagram shows that the eye-antenna disc was sectioned at different A-P positions along the D-V axis. (**B**) The cross sections (basal-apical surface) were examined by TEM. The photoreceptor (PR) and glia layers can be distinguished. (**C**–**E**) The apical side is up and basal side is at bottom. In both anterior and posterior sections, the youngest PR axons (not yet fasciculated; purple) are at the bottom (basal). The 7-axons fascicles (without the R7 axons; light green) lie just above the non-fasciculated axons. The mature 8-axons fascicles (light orange) lie at more apical positions. Scale bars are 20 μm (B) or 1 μm (**C**–**E**).

To achieve better spatial and temporal resolution in the retinotopic map analysis, we photo-activated Kaede in multiple single PR axon pairs at different A-P and D-V positions in the eye disc and then followed their axonal projections into OS and optic lamina. Kaede was expressed in the R3/4 PR by *mδ0.5-GAL4* (*mδ0.5-GAL4* + *UAS-Kaede*, abbreviated as *mδ0.5* > *Kaede*). We tested different laser intensities and different sizes of laser illumination areas to identify the optimal condition for specifically photo-converting Kaede in a single R3/4 PR pair to follow their axonal projections (Supplementary Fig. 1). Several R3/4 pairs at different locations in the *mδ0.5* > *Kaede* eye disc were labelled and examined in live or fixed discs (Fig. 1; Supplementary Fig. 2). The PR axons maintained their relative D-V and A-P positions from the eye disc to lamina (Fig. 1B–G). For example, the anterior-dorsal axon (Fig. 1B) projects to basal-dorsal position (Fig. 1C). The anterior-ventral axon (Fig. 1D) projects to basal-ventral position (Fig. 1E). In the OS, the younger (from anterior PR) axons are located at the basal position and the older (from posterior PR) axons are located at the apical positions (Fig. 1C,C’,F,F’). This order is further supported by monitoring axons in OS of the *ex vivo* cultured eye disc over time (Supplementary Fig. 3). In TEM analysis of anterior or posterior sections of eye disc (Fig. 2C,D), axons without clear fasciculation, representing young axons, are located at the basal layer, just above the basal glia layer. Apical to this are fascicles with 7 axons, representing axons from ommatidial clusters containing R8 and R1-6 but without the last differentiating R7. Further apical are fascicles with 8 axons, representing axons from mature ommatidial clusters with the full R1-8 axons. In summary, the PR axons project basally to the top of basal glia layer and the more mature axonal bundles are progressively pushed toward more apical positions in the eye disc, as well as in the OS. During this process and when projecting into lamina, their relative D-V and A-P positions are preserved.

### Young R3/4 axons transiently overshoot in lamina

**Figure 3:**
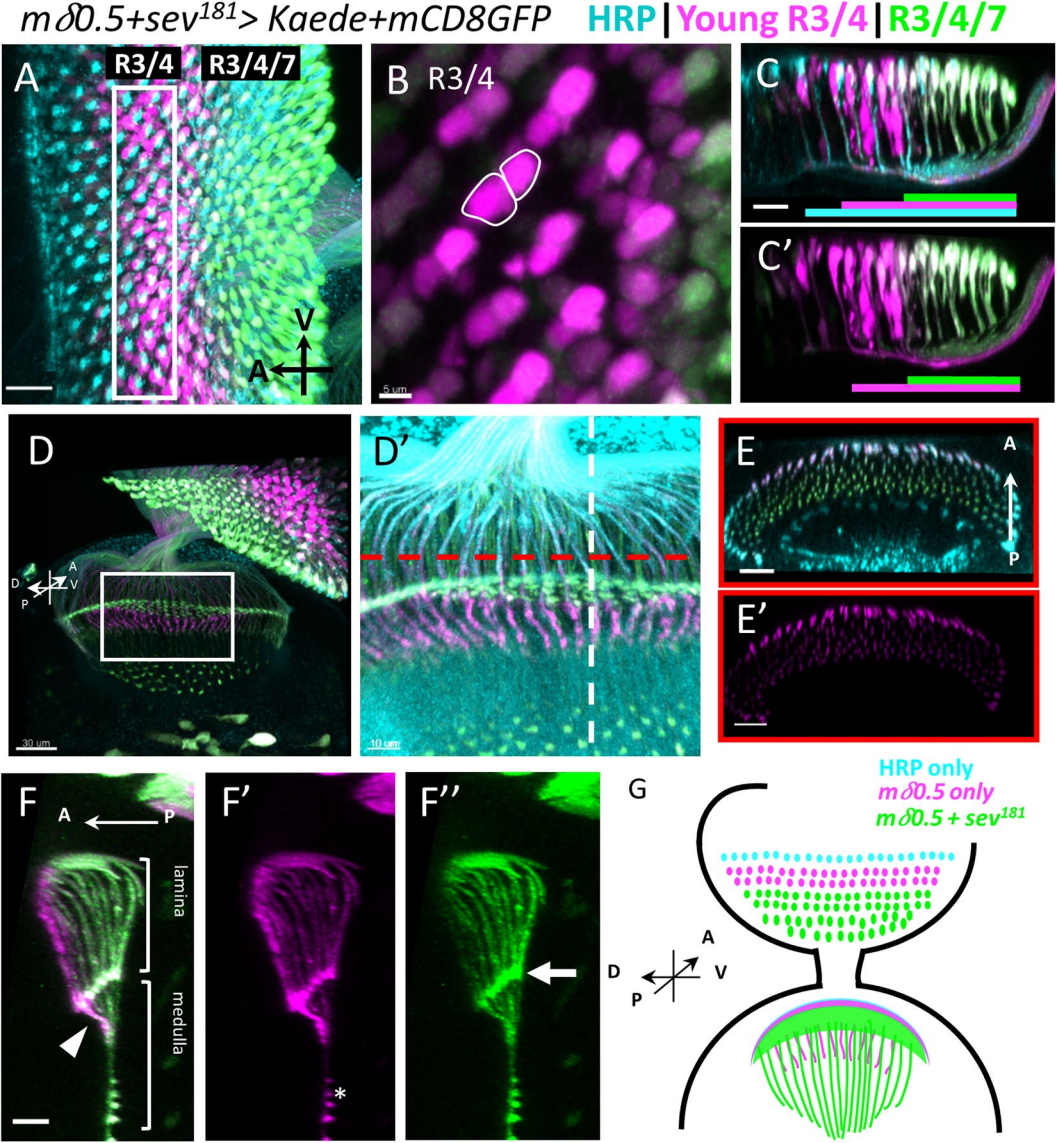
Young R3/4 axons transiently overshoot in lamina. mCD8GFP and Kaede expression are simultaneously driven by R3/4-specific *mδ0.5-GAL4* and R7-specific *sev*^*181*^*-GAL4*. Anterior is to the left for all images except (E). (**A**) HRP staining (cyan) shows all PR cell soma and axons. Young R3/4 cells are labeled by red Kaede (magenta) and mCD8GFP (green); mature R3/4/7 PRs are labeled by green Kaede and mCD8GFP. Box indicates the region of UV irradiation to convert Kaede. (**B**) R3/4 can be distinguished by cell shapes. (**C**-C’) Lateral view shows that HRP (cyan) appear before R3/4 axonal projections (red), which is anterior to R3/4/7 positive region (green). The extents of the three color signals are shown below. *mδ0.5* > *Kaede*+ *mCD8-GFP* has low level of GFP in R3/R4 that is not apparent and is not shown in the colored bars. The young R3/4 axons (red) are basal to the older R3/4/7 (green) axons. (**D**) Young R3/4 axons (magenta) project past the lamina plexus while the older R3/4/7 (green) terminate at the lamina plexus in the lamina. The overshot R3/4 axons costains with HRP (blue) (D’), indicating that these are axons. (**E**,**F**) The projections can be viewed in two optical sections (red and white dashed lines in D’) in 3D reconstruction. (**E**,E’) The young R3/4 axons (magenta) terminate in the anterior portion of lamina, while the older axons (green) terminate in more posterior regions. (**F**-F”) Lateral view of lamina shows that the overshoot young R3/4 axons (arrowhead) is at the anterior portion and past the lamina plexus (arrow) marked by the older PR axon termini. The brackets indicate the lamina and medulla regions. The asterisk indicates the background of Kaede. (**H**) Schematic drawing of eye-brain complex showing rows of R3/4 and R3/4/7 in a late thirrd instar eye disc with ~20 rows of ommatidia. The young R3/4 axons (anterior, magenta) project pass the lamina plexus, while the older R3/4/7 axons (posterior, green) terminate at the lamina plexus. Scale bar is 5 μm for (**B**), 10 μm for F-F”D, 15 μm for C-C’, 20 μm for A and E-E’ and 30 μm for (**F**).

To achieve better spatial resolution of PR axons in the lamina, we used another method to analyze the projection pattern of young versus older PR axons. PR differentiate in the order of R8 = >R2/5 = >R3/4 = >R1/6 = >R7^13^. We labeled R3/4 and R7 by expressing mCD8GFP and Kaede driven by R3/4-specific *mδ0.5-GAL4* and R7-specific *sev*^181^*-GAL4*, respectively. Since R3/4 differentiates earlier than R7, there is an anterior region (6-7 rows of ommatidia) with only R3/4 expressions, allowing specific photoconversion of the young R3/4 PRs. The anterior (younger) R3/4 are labelled by red Kaede and green GFP, while the more posterior (older) R3/4 are labelled by both green GFP and green Kaede, so the differential red-to-green ratio allows easy distinction of young vs older R3/4 axons (Fig. 3A). From the lateral view of eye disc, it is clear that the younger R3/4 axons lay at the basal layer, while the older R3/4/7 axons are located in more apical position (Fig. 3C-C’), consistent with our previous findings (Figs 1 and 2). In the lamina, the young R3/4 axons project to the anterior position (Fig. 3E).

Interestingly, we found that whereas the older R3/4/7 axons (green) terminate in a neat line (termed the lamina plexus) in the lamina (Fig. 3E) as previously reported for all R1-6 axons^15^, the younger R3/4 axons (red) terminate in deeper positions in the lamina, and even in the medulla (Fig. 3D,F-F”). Some overshooting can also be observed in *mδ0.5* > *Kaede* (Fig. 1, Supplementary Fig. 2). *mδ0.5* > *mCD8GFP* also showed similar phenomenon (Supplementary Fig. 4B). We generated MultiColor FlpOut (MCFO) clones^49^ driven by the *mδ0.5-GAL4*. In these single cell clones, the younger R3/4 axons also showed overshooting, but the older R3/4 axons terminate at the lamina plexus (Supplementary Fig. 4C). These results suggest that the young R3/4 axons transiently overshoot and then retract back to assume their final destination in lamina plexus.

We tried to ask whether this transient overshoot of R2/5 axons is a general property of all R1-6 axons. To see this early event, we need early markers for photoreceptors. The rhodopsins express only in well differentiated PR, so is not suitable for this purpose. The 24B10 stains all PR axons, but does not show any overshoot in lamina (e.g. Yu *et al*.^50^), presumably because its expression is later than the early event. *Ro-tau-lacZ* marks R2-5 axons^51^ and showed some axonal overshoot^52,53^. However, since its expression includes R3/4, whether R2 and R5 axons also overshoot is not clear. *MT14-GAL4*^54^ is reported to be specific for R2/5/8^55^, although also reported to have some expression in other PRs^56^. We found that *MT14* > *mRFP* axons do not show overshooting in lamina (Supplementary Fig. 4D). We generated MCFO clones driven by the *MT14-GAL4*. In these single cell MCFO clones, the younger PR and older PR all have axons terminate at the lamina plexus (Supplementary Fig. 4E). However, all PR cells labelled by *MT14* > *mRFP* (magenta) are also 24B10^+^, therefore the *MT14-GAL4* expression only marks older R2/5/8 axons. Therefore, whether the young R2/5/8 axons also overshoot cannot be determined using this reporter.

### WG membrane extension into optic lamina

We examined the axonal ensheathment process at single glia cell resolution by using *hs-FLP* and *repo-GAL4* to generate flp-out clones^57,58^ and monitored these clones by live imaging of *ex vivo* cultured eye disc (Supplementary Fig. 5). The glial membrane is visualized by mCD8-GFP. The WG and SG can be distinguished by their distinct morphologies. The SG can undergo cell division (Supplementary Fig. 5, yellow arrowheads). The WG progressively extends its membrane posteriorly toward the OS, presumably along the wrapped axons, with its nucleus move up and down in the cell (Supplementary Movie 1; Supplementary Fig. 5, blue and red arrowheads). We also used transmission electromicroscopy (TEM) to examine the axonal ensheathment by WG membrane. CD2-HRP^59^ was driven by the WG-specific *MZ97-GAL4* and stained by Diaminobenzidine (DAB) to detect WG membrane. In a cross section of eye disc, WG membrane can be found infiltrating to PR layer and membrane surround the PR axon (Supplementary Fig. 7AI). The WG membrane can also be noted extending into the lamina (Supplementary Fig. 7AII).

We then examined the temporal progression and the extent of WG membrane extension. Although *Mz97-GAL4* is expressed only in WG in eye discs^44^, it also expressed in some glia in the optic lobe (data not shown). Therefore, this GAL4 is not suitable for tracing retinal WG membrane extension into the optic lobe. To overcome this issue, we screened the Janelia Farm collection of *GAL4* lines with retinal glia expressions and identified a *GMR74E02-GAL4* (referred to as *WG-GA*L4 in this study) that marks only the retinal WG but no other glia in the optic lobe and brain (Supplementary Fig. 6A,B). Examination of eye discs of different developmental age showed that the progressive WG membrane extension lags behind the PR axon projection through OS to lamina (Supplementary Fig. 6D–F). The WG membrane extends into lamina but stops at the anterior layer of lamina. When all glial membrane is labelled, we noticed a region in lamina that lacks glial membrane (Supplementary Fig. 6C). The termination of WG membrane extension is not because of touching the next lamina glial membrane. Since PR axons continue to extend into the lamina (R1-6) and medulla (R7/8), there is a segment of PR axon not wrapped by glial membrane. We also generated WG MCFO clones using the *WG-GAL4*. The WG membrane extends to the lamina neurons L1-L4, but not to the lamina L5 neurons and the lamina plexus (Supplementary Fig. 7B). The younger WG membrane extends to adjacent to the lamina neurons, while the older WG membrane extend to partially surround the lamina neurons (Supplementary Fig. 7C). Consistently, recent reports showed that WG membrane progressively extend into the lamina^60,61^.

### WG membrane extension also follows the retinotopic order

In order to clearly demonstrate the A-P and D-V relationship of retinal WG and their membrane extension into lamina, we used two methods to clonally label WG cells. Both methods are based on the flp-out concept^62^. A transient heat shock during development induces the expression of *hs-Flp* to randomly induce the excision (flp-out) of a stop cassette in *UAS*-reporters in cells. The *UAS*-reporter is then responsive to GAL4 induction. In the first flp-out method, the *UAS* > *CD2, y*^+^ > *mCD8GFP*^57,58^ and *Mz97-GAL4* were used. The *hs-Flp* was induced transiently 12 hr. before late third instar larva. If the flp-out cell and its progeny cells differentiate into WG, then the *Mz97-GAL4* will drive the expression of mCD8GFP. We found that 88% of WG clones (N = 42) has only single cell, suggesting that during the 12 hr. since heat shock induction, the flp-out cell rarely undergone cell division. The advantage of this method is that live imaging is possible, but the distinction of neighboring clones is not easy. Multiple clones in a disc are numbered and the extent of their membrane, marked by mCD8GFP, can be traced. Because we never observe WG membrane extend anteriorly (Supplementary Fig. 4), the anterior extent of its membrane is interpreted as an indicator of its time and position of origin (differentiation into WG), i.e. the posterior #2 WG is older than the anterior #1 WG (Supplementary Fig. 8A). Like the PR axons, the younger WG (#1) membrane -presumably wrapping younger PR axons- is located in a more basal position in the OS than the position of older WG (#2) (Supplementary Fig. 8C,D-D’). The lateral clones #3 and #4 maintain their relative D-V positions in the OS and the medial #1 and #2 maintain their medial positions (Supplementary Fig. 8C).

**Figure 4:**
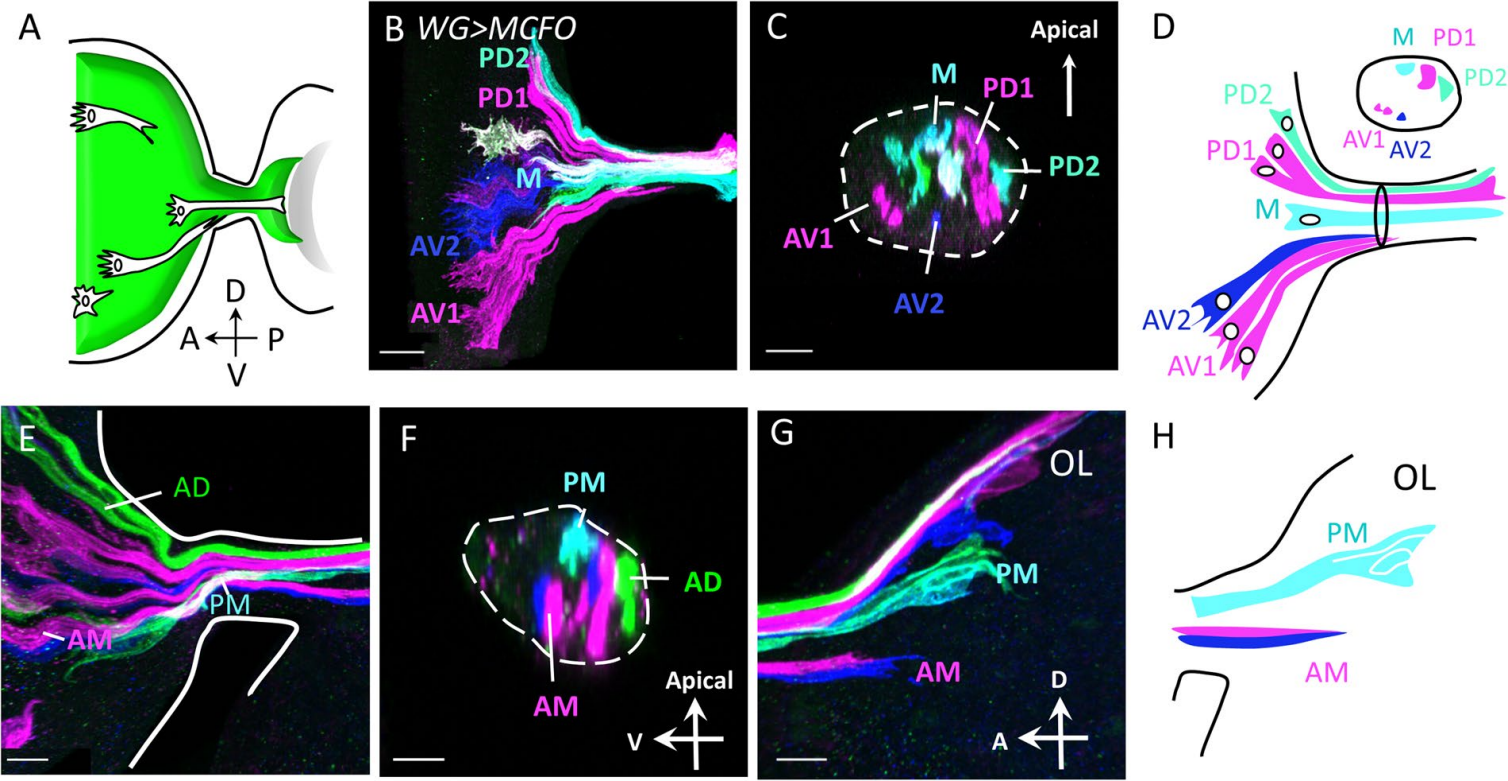
WG membrane extension follows the retinotopic rules as PR axons. MultiColor FlpOut (MCFO) clones were generated using WG-specific *GAL4* (abbreviated *WG* > *MCFO*). Anti-V5 (magenta), anti-HA (green) and anti-Flag (blue) antibodies are used to visualize membrane of WG clones. Clones are named by their relative positions along the A/P and D/V axes of the eye disc. (**A**) Schematic drawing shows the types of WG clones (white). Green indicates the extent of all WG membrane from eye disc (left) to the anterior edge of lamina (right). **(B**,**E**) *WG* > *MCFO* clones in eye discs are shown with their projections into the lamina. Dorsal is up and anterior is to left. Several *WG* > *MCFO* clones can be identified by different colors due to different reporter combinations. (**C**) An optical cross-section of the sample in (**B**) at the OS. The two anterior-ventral WG clones (AV1, AV2) show WG membrane in the basal-ventral region in OS. The two posterior-dorsal clones (PD1, PD2) have membrane in the more apical-dorsal position in OS. The medial (M) clone occupies a medial position in both A-P and D-V axes in the eye disc and in the OS. (**D**) Schematic summary of the spatial distribution of the clones in (**B**,**C**). (**E**) Among the *WG* > *MCFO* clones, an anterior-dorsal (AD), an anterior-medial (AM) and a posterior-medial (PM) clone are labeled and their membrane projections traced into the OS (F) and lamina (**G**). The AD clone occupies a basal position in the OS and has not yet reached the lamina. The AM clone occupy a basal position in OS, and has just entered the lamina. The PM clone occupies an apical position in OS and terminated in a posterior position in lamina. Dashed line outlines the OS cross section (**C**,**F**). (**H**) Schematic representation of the locations of PM and AM membrane in lamina. Scale bars are 30 μm for B, 10 μm for CEG, and 5 μm for (**F**).

The second method uses the MCFO^49^ and the *WG-GAL4* to label multiple WG clones with different combinations of the V5, HA, FLAG tags on myristoylated (membrane) GFP, as such the extent of membrane extension can be observed. This method provides better distinction of neighboring clones. The heat shock induction was 24 hr. prior to late third instar larva, therefore most clones contain multiple cells. Similar results were obtained with *Mz97-GAL4* driven MCFO analysis. WG clones preserve their relative D-V positions (Fig. 4 and Supplementary Fig. 9). In Fig. 4B, 5 WG clones were labelled: anterior WG clones (AV1, AV2) and posterior WG clones (PD1, PD2 and M). Membranes from anterior clones (younger WG) are located in the basal portion of OS, compared to those from posterior WG clones (Fig. 4C,D). The WG membrane extension follows the retinotopic rules of PRs in maintaining their D-V orders when projecting into optic lobe (Fig. 4E–H). Similar to the PR axons, the younger WG membrane extension stops at a more anterior position in the OL, while the older ones are at more posterior positions.

### WG affects PR axon projection in OS and lamina

**Figure 5:**
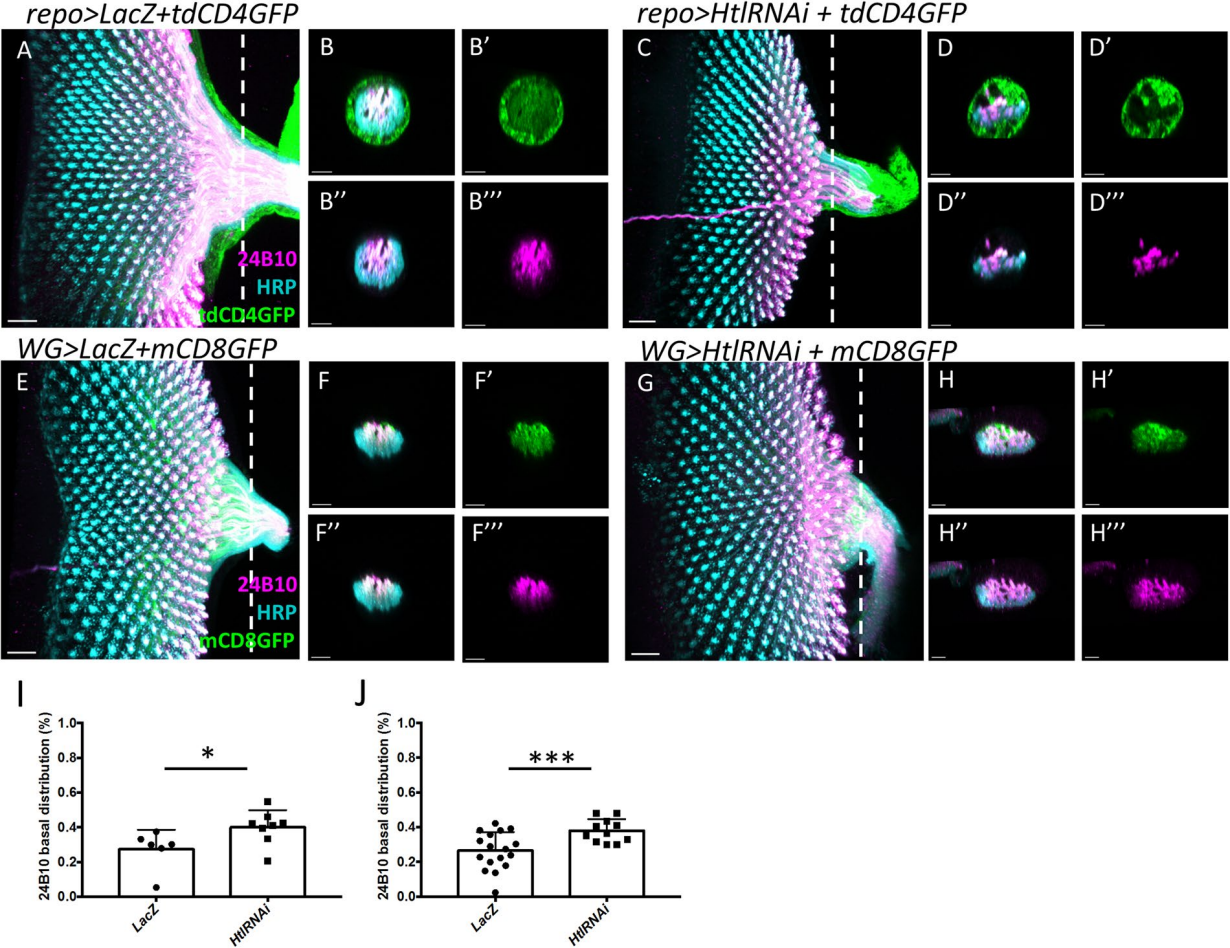
Loss of Htl in WG disrupted PR retinotopic distribution in OS. (**A**–**D**) Glial membrane is visualized by *repo* > *tdCD4GFP* (green). (**E**–**G**) WG membrane is visualized by *WG* > *mCD8GFP* (green). (**A**–**G**) 24B10 (anti-Chaoptin; magenta) stains mature PR axons. Anti-HRP (cyan) stains all PR cells and axons. The combination of 24B10 and HRP can distinguish young (HRP only) and old (both HRP and 24B10) axons. (A,**C**) 24B10 staining stays about 8 rows behind the anterior edge of HRP signal. (**B**-B”’ and **D**-D”’) Optical cross section of OS at the position of dashed lines in (**A**) and (**C**), respectively. (**B**, B’) In the control group, glial membranes occupy the entire OS cross section, with the PR axons in the central region. Younger axons (HRP only) are at the basal region, while older axons (both HRP and 24B10) are at more apical region. (**D**, D’) When *Htl* is knocked down in all glia, the glial membrane in the central region of OS is lost, indicating a loss of WG membrane. The differential apical-basal distribution of old-young PR axons is lost. (**E**) In *WG* > *mCD8-GFP*, WG membranes colocalize with axons in the central region of OS. (**F**-F”’) Younger axons (HRP only) are at the basal region, while the older axons (both HRP and 24B10) are at more apical region and colocalized with WG membranes. (**G**) When Htl is knocked down in WG by the *WG-GAL4*, the WG membrane is reduced, and the differential apical-basal distribution of old-young PR axons is lost (**H**). (**I**,**J**) Statistics for the percentage of 24B10 axonsin the basal region of OS. (**I**) *repo* > *tdCD4GFP* + *HtlRNAi*; compared with *repo* > lacZ (*p < *0.05)*. (**J**) *WG* > *mCD8GFP* + *HtlRNAi* compared with *WG* > lacZ (**p < *0.001)*. Two-tailed of Menwhitney analysis were used. Scale bar is 20 μm for (**A**–**G**).

Since the wrapping of axons is temporally tightly coupled with the extension of PR axons into the OS and lamina, we tested whether the wrapping affects the axonal retinotopic projection. In the OS, the central region consists of axons wrapped by WG membrane^41^, visualized by *WG* > *mCD8-GFP* (Fig. 5E,F). The periphery of OS consists of SG membrane^41^. FGF signaling pathway has been reported as both necessary and sufficient to promote WG differentiation^46^. In pan-glial knock down of the FGF receptor Heartless (Htl), the WG does not differentiate and there is no WG membrane in the center of the OS (Fig. 5D, compare with 5B), indicating the loss of WG membrane. The periphery of OS consists of SG membrane, so is not affected by Htl knockdown. When *Htl* is knocked down specifically in differentiated WG by the *WG-GAL4*, the WG membrane in OS is not obviously affected (Fig. 5H), although previous study showed that membrane extension into lamina is affected^47^.

**Figure 6:**
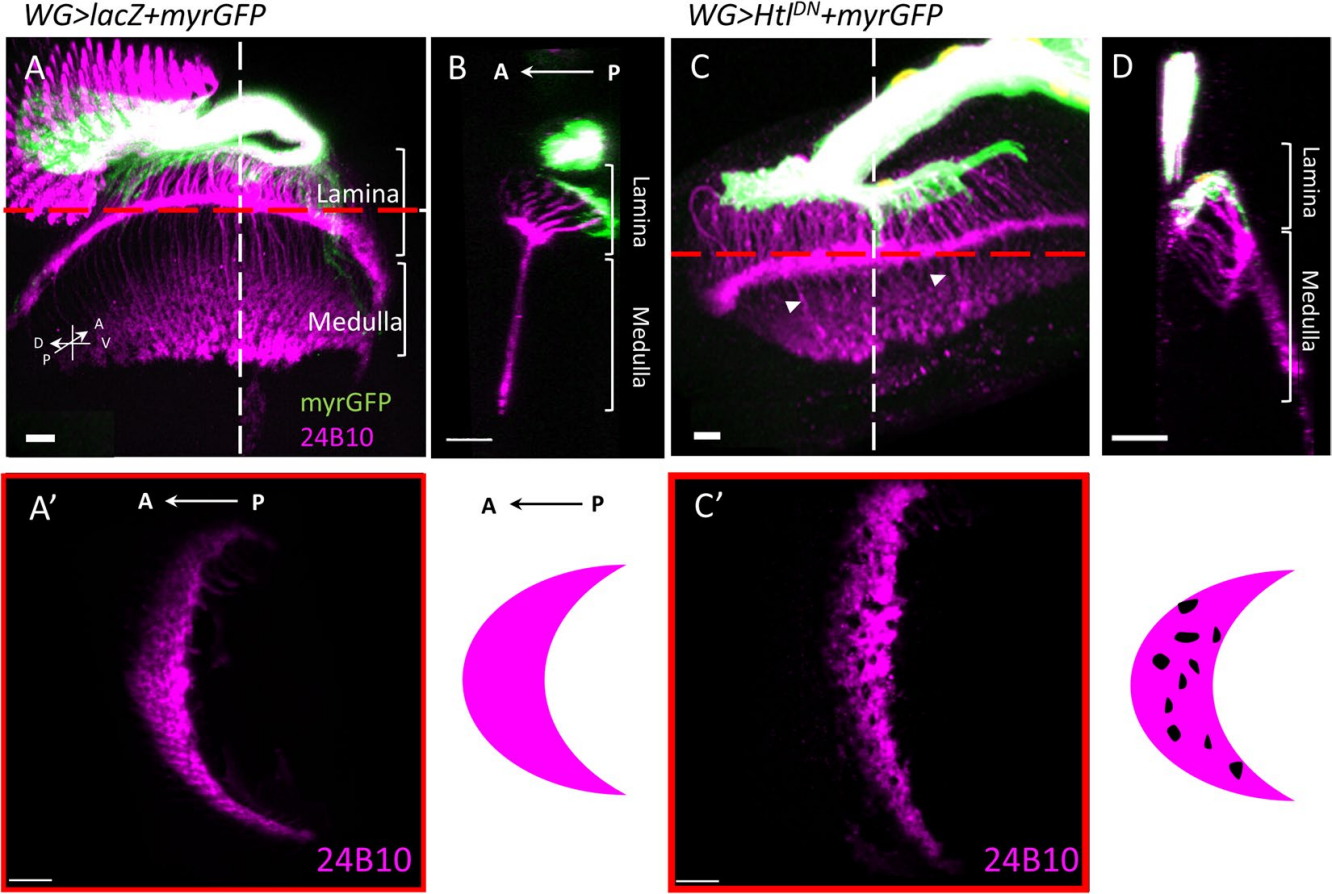
Expression of dominant-negative Htl in WG resulted in aberrant PR projection pattern in lamina. (**A**–**D**) WG membranes are visualized by *WG-GAL4*-driven mCD8GFP. 24B10 stains older PR axons. (**A**) In the control sample, R1-6 axons terminate in the lamina plexus and the R8 axons extend into the medulla. (**B**) An optic section lateral view along the white dashed line in (**A**). (**C**,**D**) WG-specific expression of a dominant-negative *Htl* (*WG* > *Htl*^*DN*^). The retinotopic projection of PR axons (A’, C’) are viewed from optical section indicated by red dashed line in (**A**, **C**), respectively. (A’, C’) The anterior border of lamina is to the left. (**C**) In *WG* > *Htl*^*DN*^, the WG membrane border in the anterior region of lamina becomes irregular. The PR axon termini at the lamina plexus is less compact than control. (**D**) In a lateral view, the lamina plexus is disorganized. (C’) Small gaps are found in the PR axon field in lamina, suggesting irregular PR axon projections. Scale bars are 20 μm for AA’BC’D and 10 μm for (**C**).

The young and old PR axons are distinguished by the relative timing of 24B10 and HRP staining^22^. HRP-only axons (cyan) represent younger axons, while 24B10-positive axons (magenta) represent older axons (Fig. 5). In wild type, about 25% (N = 6 for *repo-Gal4* control and N = 17 for *WG-Gal4* control) of the 24B10-positive axons are in the basal volume. The percentage increased to 39**%** in pan-glial Htl knockdown (N = 8) and 38% in WG-specific Htl knockdown (N = 11) (Fig. 5I,J). The 24B10 + axons in the Htl knockdown OS also appears to be more dispersed than in controls (Fig. 5I,J). In mutant animals, axons layers become extremely flattened. In addition, young (HRP staining only) and old (24B10) axons were patchily distributed. Additionally, WG membrane extends into the OS and lamina, but terminate in lamina in an irregular manner (Figs 5F’,H’ and 6C,D). Same defects of membrane extension were previously reported while overexpressed *Htl*^*DN*^ driven by *Mz97-GAL4*^47,48^. In the lamina, when dominant-negative Htl was expressed in WG, the lamina plexus is less compact than in wild type (Fig. 6C,D, compare with 6A,B). A projection from a different angle shows that the *WG* > *Htl*^*DN*^ lamina has some gaps in the lamina plexus (Fig. 6C’, compare with Fig. 6A’). These results showed that the reduction of Htl in WG affects PR axon retinotopic projections in OS and lamina.

**Figure 7:**
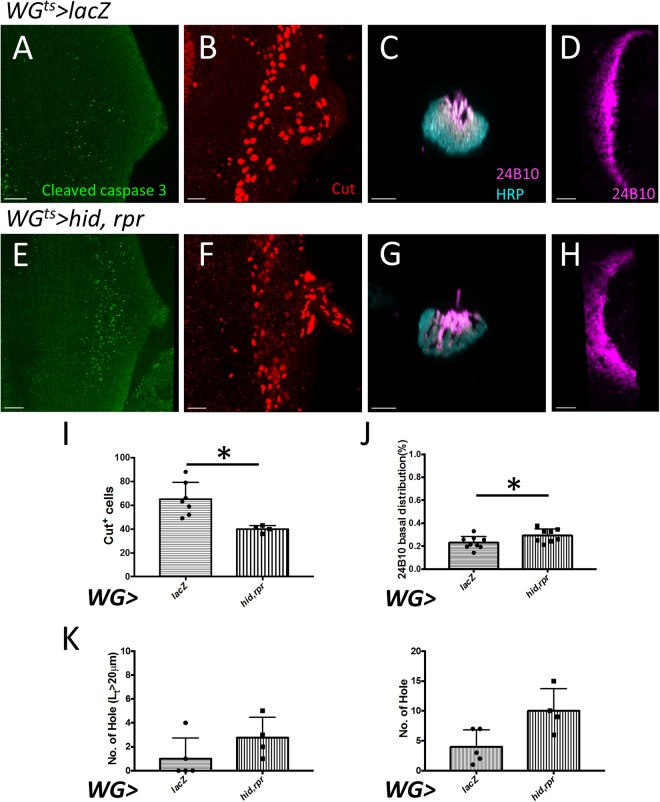
Killing of wrapping glia caused defect in retinotopic projection. The death genes *hid* and *rpr* were expressed by *WG-GAL4* with *tub-GAL80*^*ts*^ (*WG*^*ts*^ > *hid* + *rpr*). (**A**–**D**) *WG*^*ts*^ > *lacZ* served as control. (**E**–**F**) After 12 hrs temperature shift to 30 °C to induce death genes expression, the eye-brain complex is examined immediately. The basal WG layer showed significant increase of the apoptotic marker cleaved caspase 3 signals (E, green) and decrease of Cut^+^ WG cell number (F, red; quantitative analysis in I). Small Cut^+^ puncta, in contrast to the nuclear staining and probably representing debris of apoptotic WG cells can be detected in the middle region of the *WG* > *hid* + *rpr* eye disc (**F**). (**C**,**G**) In the optic stalk, the younger axons (24B10 alone, magenta) showed differential apical localization of in the control group (C), but in *WG* > *hid* + *rpr* are more disorganized in the optic stalk with more basal localization (G; quantitative analysis in J). (**D**,**H**) In the optic lamina, the retinotopic projections in lamina plexus are more disorganized in *WG* > *hid* + *rpr* (**H**) as compared with the control group (D). (**K**) Quantification data of the number of holes and the number of large holes (arbitrarily defined as holes with perimeter L_t_ > 20 μm). Scale bars are 20 μm for all.

We examined the effect of WG on PR retinotopic projection by other manipulations. We first killed WG by expressing the pro-apoptotic genes *head involution defective* (*hid*) and *reaper* (*rpr*) using *WG-GAL4*. Since the continuous expression of death genes in *WG* > *hid* + *rpr* lead to larval death, we used *tub-GAL80*^*ts*^ to temporally control the death genes expression (abbreviated as *WG*^*ts*^ > *hid* + *rpr*). After a 12 hr. induction of hid and rpr expression in late third instar larvae, there is significant increase of the apoptotic marker cleaved caspase 3 (Fig. 7A,E) and significant decrease of Cut+ cells in the eye disc (Fig. 7B,F), indicating the killing of a significant proportion of WG cells. The preference of younger PR axons to occupy the more apical region in OS is reduced (Fig. 7C and G). In the lamina, there is more and larger holes (Fig. 7D,H), suggesting a more disorganized retinotopic distribution. These results demonstrate that WG is important for the proper retinotopic projection of PR axons. When CG is similarly killed (in *C135*^*ts*^ > *mCD8GFP* + *hid* + *rpr*), the number of WG is slightly reduced, the PR retinotopic projection pattern in OS is not affected, but there are more holes in the lamina (Supplementary Fig. 10G–J). When *hid* were driven by the SG-specific *C527-GAL4*, the number of WG was not affected. Although WG is derived from SG^44^, the 12 hr of *hid* induction was too short to significantly affect the transition from SG to WG. In *C527*^*ts*^ > *hid*, the PR retinotopic projection pattern in OS and in lamina were not significantly affected (Supplementary Fig. 10O-R). These results showed that the WG has a major role in PR retinotopic projection in OS and in lamina.

We also blocked WG membrane extension to test for the effect on PR retinotopic projection. The Borderless (Bdl) receptor is expressed in WG to receive the signal Turtle (Tutl) expressed in the PR axon^47,48^. We knocked down Bdl in WG (*WG* > *mCD8GFP* + *Bdl-RNAi*). As previously reported^47^, Bdl knockdown reduced WG membrane extension in the lamina (Supplementary Fig. 11D-D’). The retinotopic projection pattern in OS is not affected (Supplementary Fig. 11B,D), probably because the WG membrane in OS was not affected. The retinotopic projection in lamina was disturbed (Supplementary Fig. 11C,F). All of these results indicate that the WG, especially its membrane, plays a role in the retinotopic projection of PR axons into the optic lamina.

## Discussions

### Retinotopic map preserves the spatial information from retina to OS and lamina

In this study, we focus on the retinotopic map from retina to lamina. Our single-cell analyses of PR showed that the relative A-P and D-V positions are preserved from the retina to the OS and to the lamina, confirming previous findings^22^. The D-V axon guidance information is provided by the gradient of the DWnt4 expressed in ventral lamina and the receptor Dfrizzled2 along with its downstream signaling component disheveled in the retinal axons, respectively. The dorsal-specific transcription factor Iroquois acts in dorsal PRs to attenuate the response to DWnt4^30^. However, lamina DWnt4 gradient is unlikely to act in the OS. Therefore, the maintenance of the retinotopic map in the OS may rely on as yet unidentified guidance system or on the interaction among neighboring axons.

### Transient overshoot of young R3/4 axons in lamina

**Figure 8:**
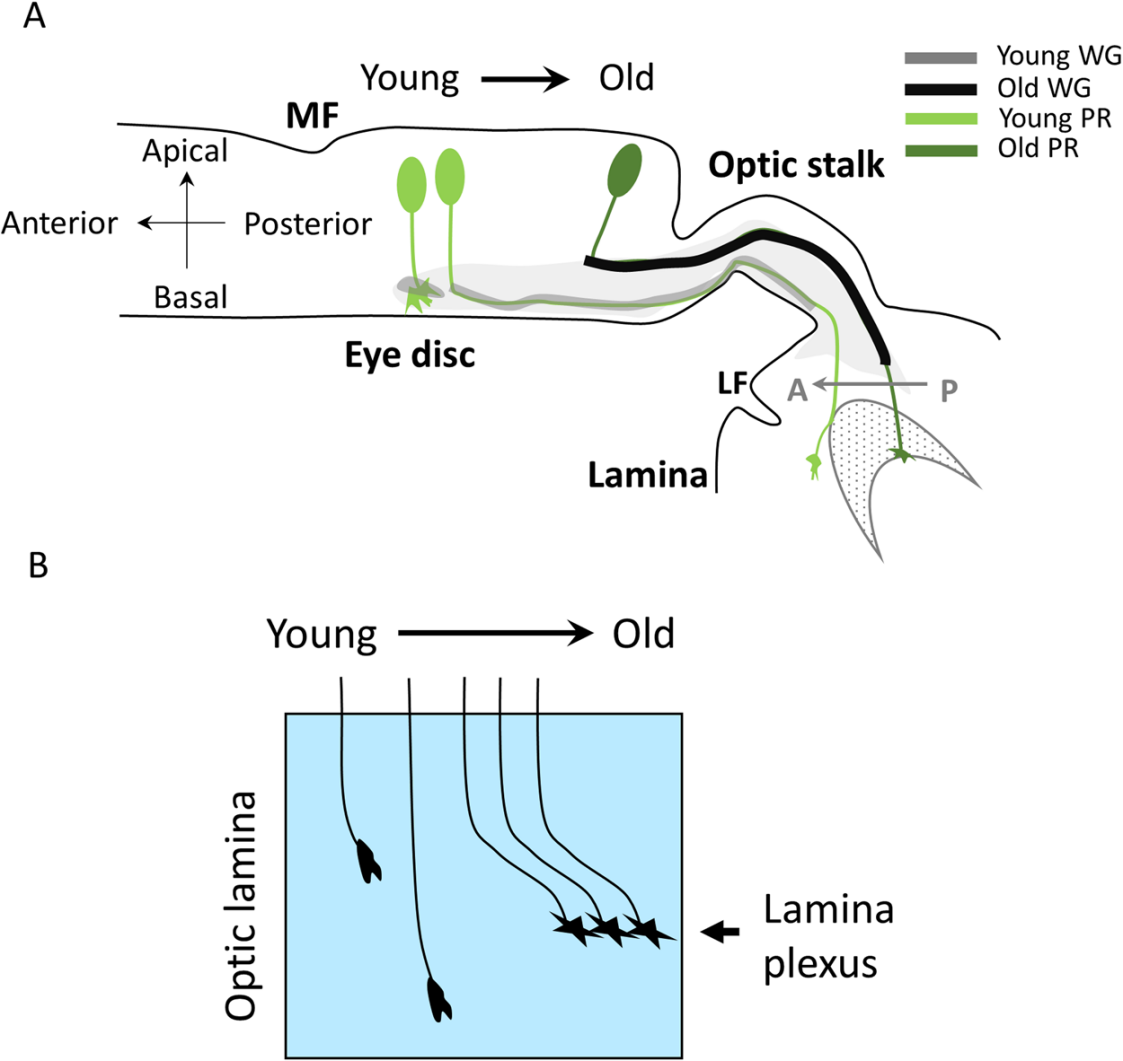
Summary of spatial and temporal correlation between PR and WG retinotopic projections. (**A**) Schematic illustration of the progressive WG membrane extension and the temporal and spatial relationship with the PR axon extension into OS and lamina. (**B**) Schematic illustration of the R3/4 axons projection over time. The youngest R3/4 axons is depicted to the left and is extending their axons into lamina. The axons overshoot past the lamina plexus. More mature R3/4 axons terminate at the lamina plexus.

R1-6 PR axons terminate in the lamina plexus between the layers of epithelial glia and marginal glia^15,36^. Such termination of R1-6 axons in lamina is dependent on the interaction between the PR axons and lamina glia^36–38^. Unexpectedly, we found that the young R3/4 axons overshoot and pass the lamina plexus to medulla (Fig. 3). This is not observed in older R3/4 axons, suggesting that the overshoot is a transient event (Fig. 8B). This transient axonal overshoot has not been reported before. One possible reason is that previous studies have used 24B10 as a marker for all PR axons^22^. 24B10 is expressed later than the *mδ0.5-GAL4* and thus only in older PR axons, therefore the earlier overshoot event was not observed. We tried to test whether all R1-6 axons transiently overshoot but have not obtained conclusive result. Specific markers for R1/2/5/6 that are expressed early are needed. The R2/5/8-specific *MT14-GAL4* unfortunately marks only more mature R2/5/8 (Supplementary Fig. 4).

R1-6 overshoot phenotype has been observed in other situations. Several transcription factors including *Brakeless* (Bks), *Runt* (Run), *Sequoia* (Seq), Hindsight (Hnt) (and its target genes *tiggrin* (*tig*), *jitterbug* (*jbug*)/filamin, *off-track* (*otk*)), transcriptional coactivator and phosphatase Eyes absent (Eya), receptor tyrosine phosphatase PTP69D, the Jak/STAT pathway and the kinase Pelle are all required for proper lamina termination of R1-6 axons^23,55,63–67^. Whether these genes act on the retraction of the transiently overshot axons or changed the targeting specificity from lamina to medulla is not clear.

Glia in the target field also plays a role in the R1-6 termination in lamina. In *glia cell missing* (*gcm*) and *gcm2* double mutant, lamina gliogenesis is affected^38^. In addition, ubiquitin protease Nonstop (Not) is required for the migration of the epithelial, marginal and medulla glia to their proper positions in lamina^36^. Lamina glia migration can be affected by the JAB1/CSN5 subunit of the COP9 signalosome complex acting in PRs^37^. Mutations or malfunction of these molecules all affected lamina glia and resulted in the R1-6 overshoot phenotypes, suggesting a role of lamina glia in R1-6 axon targeting. Whether the transient overshoot and retraction of R3/4 axons is also regulated by lamina glia is not clear.

### WG is important for the retinotopic map formation

Lamina glia are critical for the proper R1-6 axon termination in lamina to form the lamina plexus^36–38^. Whether the retinal basal glia affects retinotopy is not known. Here we show that WGs in eye discs non-autonomously affect the retinotopic projections of PR axons in OS and in lamina. When Htl is reduced, the apical-basal distribution of old-young PR axon projection in the OS is disturbed, and the proper termination of R1-6 axons in the lamina and the formation of lamina plexus is also disturbed. Since the retinal WG membrane does not extend to the lamina plexus, how do WG affect the R1-6 termination at lamina plexus presents an interesting question. Recent study has shown novel ability of WG to synchronize the differentiation of lamina neuron through the secretion of insulin-like peptides^60^. It is possible that WG affects the R1-6 termination at lamina plexus by secreted molecules.

## Methods

### *Drosophila* stocks

Fly stocks are maintained at room temperature (RT). For all experiments, the flies are raised at 25 °C unless otherwise indicated. Special conditions were indicated individually. Fly stocks from Bloomington Stock Center are: *repo-GAL4* (BSDC#7415)^68^*; mδ0.5-GAL4*^69^ (BSDC#41782 and #41795, on chromosome II and III, respectively); *GMR74E02-GAL4* (BSDC #48320); *hsFLP; UAS* > *STOP* > *myr-smGdP-HA UAS-* > *STOP* > *myr-smGdP-V5 UAS* > *STOP* > *myr-smGdP-FLAG* (BSDC #64085)^49^; *Mz97-GAL4*^44^; *hsFLP*122*; USA* > *STOP* > *mCD8GFP (III)*^57,58^; y1 w*;*.UAS-htl-DN; UAS-htl.DN (*BDSC#5366)^70^; *MT14-GAL4*^54^ (BDSC#37293); *C135-GAL4*^44^ (BDSC#*6978); C527-GAL4*^44^; *UAS-hid; tub-GAL80*^*ts*^*(II) (*BDSC#7019). *UAS-Bdl-RNAi (V4806)* is from VDRC and also gift from Dr. Yong Rao. *sev*181*-GAL4* (kindly provided by Chi-Hon Lee)^71^; *Htl-RNAi* (kindly provided by Christian Klämbt)^46^; *UAST-myr-GFP-V5-P2A-H2B-mCherry-HA* is an unpublished gift from Yung-Heng Chang and Joshua Dubnau (Department of Anesthesiology, Stony Brook University); *UAS-Kaede.K22* (kindly provided by Ann-Shyn Chiang)^33^; *UAS-hid, UAS-rpr* (gift from Suewei Lin); *UAS-CD2-HRP* (kindly provided by Tzu-Yang Lin); *Ro-tau-lacZ* (kindly provided by Philip A. Barker).

### Clonal induction

*Mz97-GAL4* flp-out clones were generated using *hs-FLP122; Mz97-GAL4; UAS* > *CD2* > *UAS-mCD8GFP*. MCFO clones were induces by *hs-FLP*^*G5*^ and driven by *GMR74E02-GAL4*. In both experiments, larvae of mixed ages were heat-shocked for 10 minutes in 38 °C water bath. Larvae were raised for another 12 hr. (for *Mz97-GAL4* flp-out clones) or 48 hr. (for MCFO clones) after the heat-shock and larvae at late third larval stage were dissected to examine the clones. The glial flp-out clones were generated by heat shock of *repo* > *hs-Flp*^*122*^*;*+*/*+; *UAS-mCD8-GFP* for 15 min, and the eye disc was dissected 24 hr. after heat shock for *ex vivo* culture.

### Conditional inactivation of GAL80^ts^

The flies after mating were raised at 17 °C (permissive temperature). When 12 hr before late 3^rd^ larval stage, the larvae were shifted to a nonpermissive temperature (30 °C) to induce death genes expression for the indicated time.

### Immunohistochemistry and confocal microscopy

The eye-brain complex was dissected from late third instar larvae and fixed in 4% EM-grade paraformaldehyde (PFA, Electron Microscopy Sciences, 30525-89-4) in 1X PBS (phosphate buffered saline) for 20-25 minutes and washed three times in 1xPBS. Primary antibodies were: mouse anti-2B10(CUT) (DSHB, 1:50); mouse anti-24B10^27^ (DSHB, 1:200), anti-HA (4C12 mouse Ab, Abcam ab1424; 1:200), anti-FLAG (anti-dykddddk rabbit Ab, Sigma Aldrich, F2555; 1:200), anti-V5 (rat Ab, Novus Biologicals #NBP1-06712; 1:200) and rabbit anti-Cleaved Caspase-3 (Asp175, 1:200, Cell Signaling). Secondary antibodies used were Alexa-488, Cy3, Alexa-647 and HRP-Alexa-647(Jackson Immunoresearch, mouse and rat minimal cross-talk versions). All antibodies were diluted by PBST with 10% (v/v) normal goat serum (Jackson Immunoresearch). All images of fixed samples were acquired by Zeiss LSM 780 with the Plan-Apochromat 40x/1.4 oil objective. For live imaging and UV photoconversion of Kaede, LSM 710 inverted microscope was used with C-Apochromat 40x/1.2 W korr objectives. Optical sections were 0.2 (for 24B10/HRP staining), 0.8 and 1.2 μM thickness for fixed and live samples, respectively.

### Image processing and analysis

3D images were reconstructed and analyzed by the commercial software IMARIS 8.4.1 or 9.0.0 (Bitplane, Germany). XY figures are all presented as whole Z-stacks processed by IMARIS unless otherwise indicated. Cross and longitudinal sections were shown in partial Z-stack projections (2.5–10 μM). For live imaging, images were recorded every 10 minutes for 10–16 hrs. To analyze axonal movement, images were cropped into smaller region that only covers OS. IMARIS threshold cutoffs of 13.5 and 7.5 were used for green channel and red channel respectively to get rid of global background signals and make the outline of the OS clearer. The “Measurement points” function was used to define the length from top to bottom and from top to center of axons in OS around 40 time points. To analyze 24B10 distribution in optic stalk in Fig. 5, OS sections were cropped by IMARIS. Next, volumes of 24B10 and HRP staining were obtained after 3D surface rendering. The region covering the OS was then divided equivalently along Z axis. Percentage of 24B10 volume in apical region and basal regions were recorded. To analyze the size of hole in Fig. 7, the perimeter of the holes was measured.

### *Ex vivo* tissue culture and confocal microscopy

Eye-brain complexes were cultured *ex vivo* as described previously^45,72^.

### Transmission electron microscopy (TEM)

The larvae were cut into halves by dissecting scissor to reduce the sheared damage on the tissues. The head cuticle was gently removed by scissor to expose the eye-brain complex. The dissected eye-brain complex was quickly fixed in fixative containing 4% paraformaldehyde and 2.5% glutaraldehyde in 0.1 M sodium cacodylate (Sigma), pH7.4 in 4 °C for overnight. The tissues were washed three times (15 minutes each) with 0.1 M sodium cacodylate, 2% OsO4 (Electron Microscopy Sciences) in 0.1 M sodium cacodylate in RT for 1 h, three times in Milli-Q water and switched to 2% uranyl acid (Polysciences) for 30 mins in RT, then washed by Milli-Q water, and dehydrated by a series of different concentration of ethanol (50% once, 70% once, 80% once, 90% once, 95% once and 100% three times, propylene oxide twice; 15 minutes for each series dehydration). The dehydrated tissues then underwent a series of resin replacement in increasing ratio of Epon (EMbed-812 Kit)/propylene oxide (SIGMA-ALDRICH #471968) (1:3, 2:1, 1:1, 1:2, 1:5, then pure Epon for three times). In the final step, individual eye-brain complex was embedded in resin. Resin blocks were baked in 60 °C oven for 48 hrs. 80 nm-thick sections were acquired with a diamond knife (Ultracut, Reichert-Jung, Vienna, Austria) and examined by TEM (Tecnai G2 Spirit TWIN, FEI Company, Hillsboro, OR) equipped with a Gatan CCD Camera (794.10.BP2 MultiScanTM). For visualization of HRP-expressing WG, Diaminobenzidine (DAB) staining are as described^73^ but without Ni-intensified.

### Photoconversion of Kaede

The photoconvertible fluorescent protein, Kaede^31–33^ was used in this study. UV irradiation induces a peptide cleavage resulting in the rapid and irreversible conversion from green fluorescence to red fluorescence^32^. The red signal slowly decays, while the green signal slowly recovers, probably due to newly synthesized Kaede protein. The green-to-red photoconversion was induced by 405 nm laser beam with 1.5–7% power of 25 mW laser (LASOS lasertechnik GmbH, LGN3001) for 300 hits (over 4.53 s; 0.01513 s/hit; without interval). The irradiating conditions were optimized (Supplementary Fig. 1). Low laser power and longer exposure work well in single PR irradiation. In order to reduce photoconversion in non-target cells, we only irradiated a single PR pair in a region smaller than 3 × 3 pixel^2^. The bleaching mode in Zen software was used to draw region of interests (ROI). Once the red-to-green ratio was raised, the activated region could be distinguished until the end of imaging period (9–16 hr). Due to the background photoconversion during dissecting and mounting, the red signals are clearer when overlaid with green signals.

## Electronic supplementary material

Supplementary Information

Supplementary Movie 1

Supplementary Movie 2
