# Supplementary material for: Temporal and spatial order of photoreceptor and glia projections into optic lobe in Drosophila
Source: Sci Rep. 2018 Aug 23;8:12669. doi: 10.1038/s41598-018-30415-8 (PMC6107658; doi:10.1038/s41598-018-30415-8)
Supplement: Supplementary file 1 — Supplementary Information [file 41598_2018_30415_MOESM1_ESM.docx]

**Temporal and spatial order of photoreceptor and glia projections into optic lobe in *Drosophila***

Yen-Ching Chang^1,2^, Chia-Kang Tsao^1,2^, Y. Henry Sun^1,2 *^

^1^ Institute of Genomic Sciences, National Yang Ming University, Taipei, Taiwan

^2^ Institute of Molecular Biology, Academia Sinica, Taipei, Taiwan

*Corresponding author: mbyhsun@gate.sinica.edu.tw


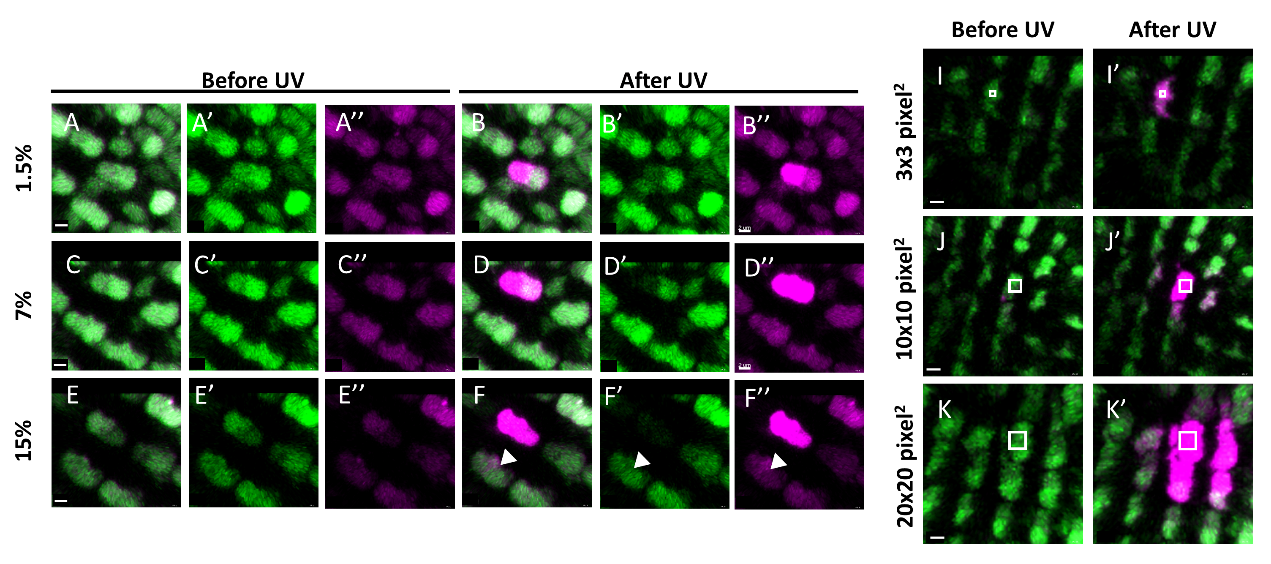


**Supplementary Figure 1. Defining the optimal condition for Kaede photoconversion.** Kaede is expressed in the R3/4 PR by the *mδ0.5-GAL4*. (A-F) Different laser powers were tested to photo-activate Kaede. Under 1.5% (A, B), 7% (C, D) and 15% (E, F) power of UV laser (25mW laser, LASOS lasertechnik GmbH, LGN3001) with fixed induction region (3x3 pixel^2^) on a single pair of R3/4 PR. (A, C, E) Kaede signal before UV irradiation. (B, D, F) Kaede signals after UV irradiation. (B) The GFP (green) signal is not completely converted to the red signal after UV treatment. (D) More red signals are induced but there is residual green signal, indicating incomplete photoconversion. (F) The green Kaede is completely photoconverted into red Kaede. However, weak red Kaede signal (arrowhead) can also be observed outside of the single R3/4 being activated, indicating a spillover effect. (I-K) Three different size of the photoactivated areas, 3x3, 10x10, 20x20 pixel^2^, were tested. (J’, K’) Larger photoactivation area causes undesired spillover activation of neighboring ommatidia, so the 3x3 pixel^2^ area is used for subsequent experiments. Scale bar is 2μm (A-F) and 3μm (I-K).


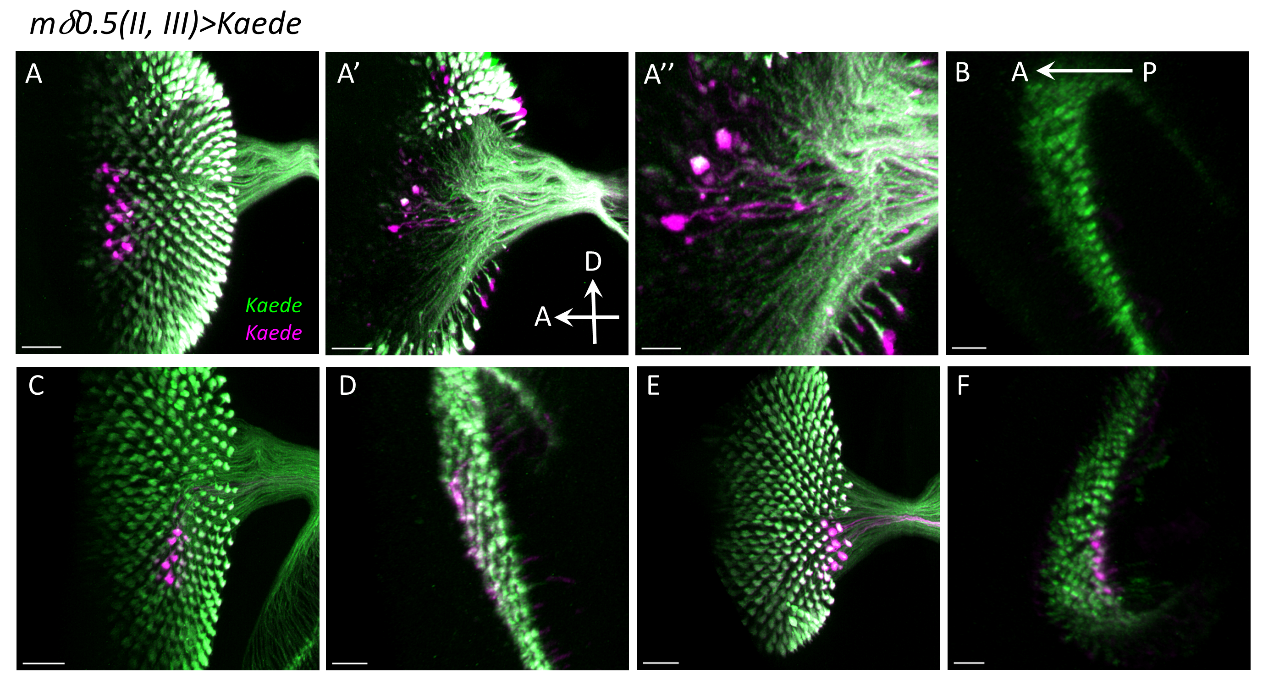


**Supplementary Figure 2**. **Progressive retinotopic projection of PR axons into lamina.** (A-F) We examined axons projection in fixed samples. *Kaede* expression in R3/4 is driven by two copies of *mδ0.5-GAL4*. Whole Z-projections in (A, C and E) indicate single groups of PRs irradiated in anterior-most, anterior and posterior-most regions respectively. Anterior is to the left in all images. (A-A’’) Photo-activated PRs undergo axonal extension, as demonstrated in Z-projection of the basal layer (A’-A’’). In this sample, the photoactivated axons has not reached the optic lobe (B). (C and E) Axons from anterior and posterior-most PR terminate in the anterior (D) and posterior rim (F) of lamina. Scale bars are 20μm for AA’CE, 10μm for A’’BDF.


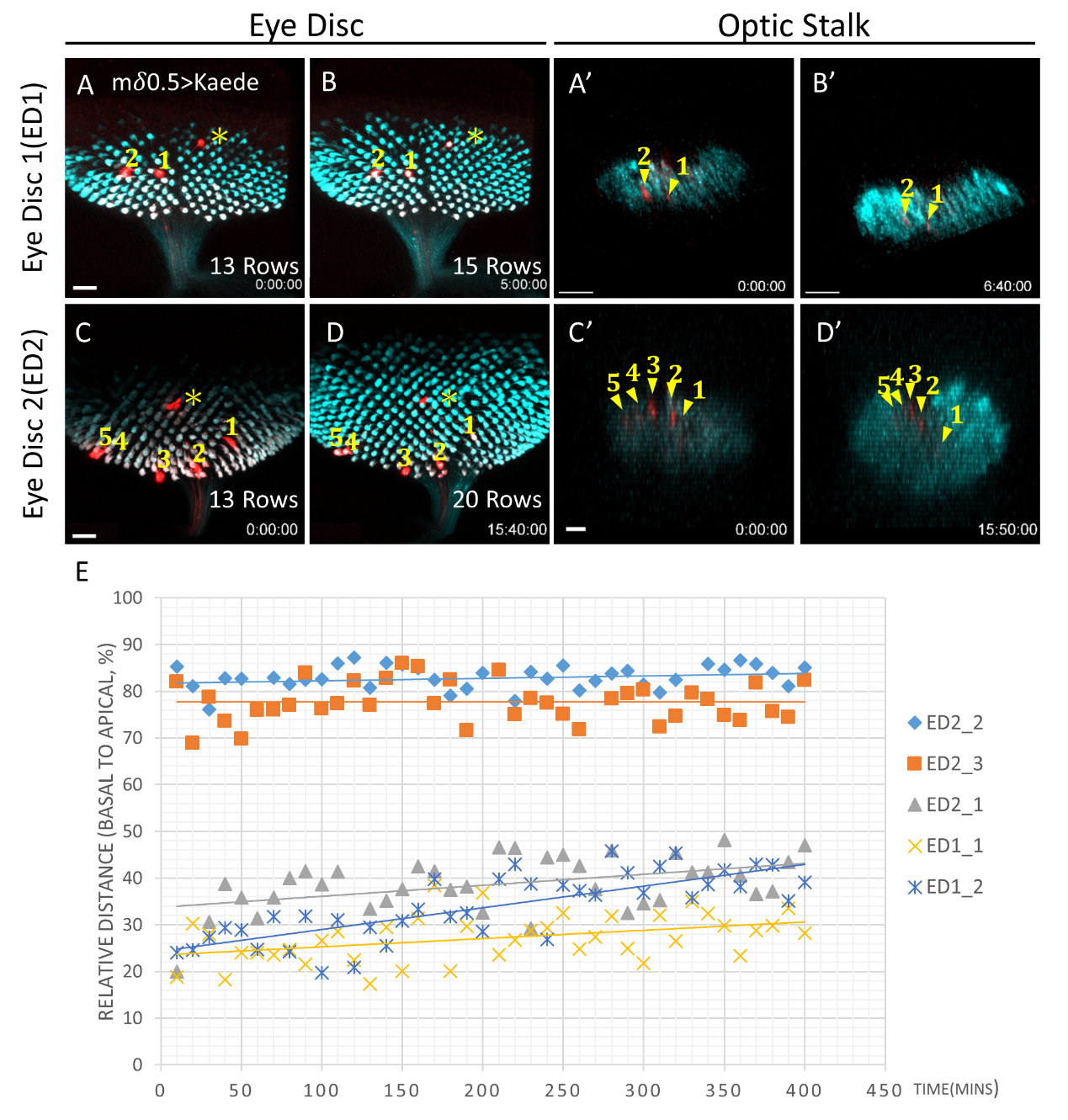


**Supplementary Figure 3. The A-P and D-V positions of PR axons are conserved in the OS.** Kaede expression in R3/4 is driven by *mδ0.5-GAL4.* The green Kaede in selected single R3/4 pairs were photoconverted to red. Two eye discs and 7 pairs of R3/4 axons projecting into OS were imaged in *ex vivo* cultured eye disc and analyzed. (A-B) The first disc grew from 13 rows (A) to 15 rows (B) of ommatidia in 6.5 hours. (C-D) The second disc grew from 13 rows to 20 rows in 16 hours (C and D). Individual axons that can be traced into OSs were numbered separately in two eye discs. The asterisk labels young PR in anterior and its axon has not reached the OS. (A’, B’) 2 axons from anterior PR (#1-2) were traced. (C’, D) In the second example, 5 PRs can be tracked in to OS. Only three axons were analyzed (1 axon from anterior PR and 2 axons from posterior PR (#1-3). (E) The relative basal position of an axon in OS is calculated as (distance between apical top to axon)/ (apical-basal distance of OS). All three anterior axons (ED1-1, ED1-2 and ED2-1) showed weak positive correlation (R² = 0.20561; R² = 0.5953; R² = 0.16855), suggesting that basal axons slowly move up to apical portion in OS. Posterior axons (ED2-2 and ED2-3) (R² = 0.04489; R² = 2.3E-05) stay consistently in the apical region. Scale bars are 20μm for A-D and 10μm for A’-D’.

**Supplementary Movie 1.** **PR axons in optic stalk gradually move from basal to apical position during development.** The optical section of optic stalk was from ED2 in Sup Fig. 3. Axons were labeled by photoconverted red Kaede. The positions of axons were recorded every 10 mins for 16 hrs.


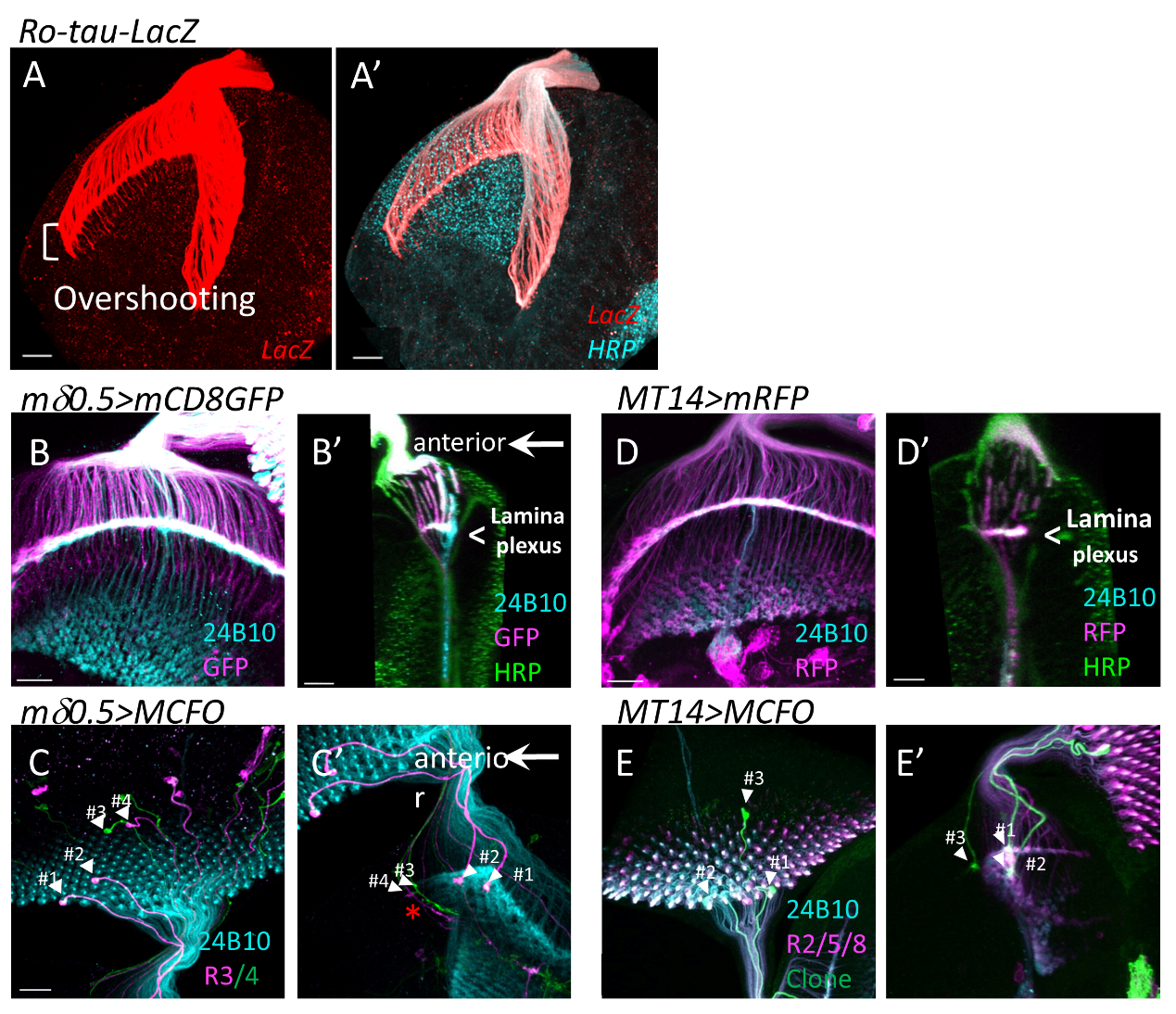


**Supplementary Figure 4. R3/4 axons transiently overshot lamina plexus in lamina.** (A, A’) *Ro-tau-lacZ* (red) labels R2-R5 axons in optic lobe. White bracket indicates axonal over-extension that bypass the lamina plexus. HRP (cyan) marks all axons. The R3/4-specific *mδ0.5-GAL4* (B) and R2/5/8-specific *MT14-GAL4* (D, E), were used to drive mCD8GFP (magenta in B, B’) or mRFP (magenta in D-D’), respectively. HRP staining (green) show all PR cell soma and axons. 24B10 (cyan) stains older PR cell soma and axons. (B, B’) Over-extension can be observed in younger R3/4 axons (magenta only) in XY plane and in XZ optical section. (C, C’) Several MultiColor Flip-Out (MCFO) clones, generated by the R3/4-specific *mδ0.5-GAL4*, in the same eye disc allowed comparison of younger (#3 and #4, 24B10^-^) and older (#1 and #2, 24B10^+^) PR axons. The younger #3 and #4 axons showed over-extension in lamina (highlighted by asterisk in red). The older #1 and #2 axons terminate in the lamina plexus. (D, D’) In contrast, no obvious over-extension can be found in R2/5/8–axons labelled by *MT14>mRFP* (magenta) shown in different optical sections. (E, E’) In MCFO clones generated by the R2/5/8-specific *MT14-GAL4*, the younger PR (#1) and older PR (#2 and #3) have axons terminate at the lamina plexus. Since all R2/5/8 cells labelled by *MT14>mRFP* (magenta) are also 24B10^+^, the *MT14-GAL4* expression only marks older R2/5/8. Scale bars are 20μm for all.


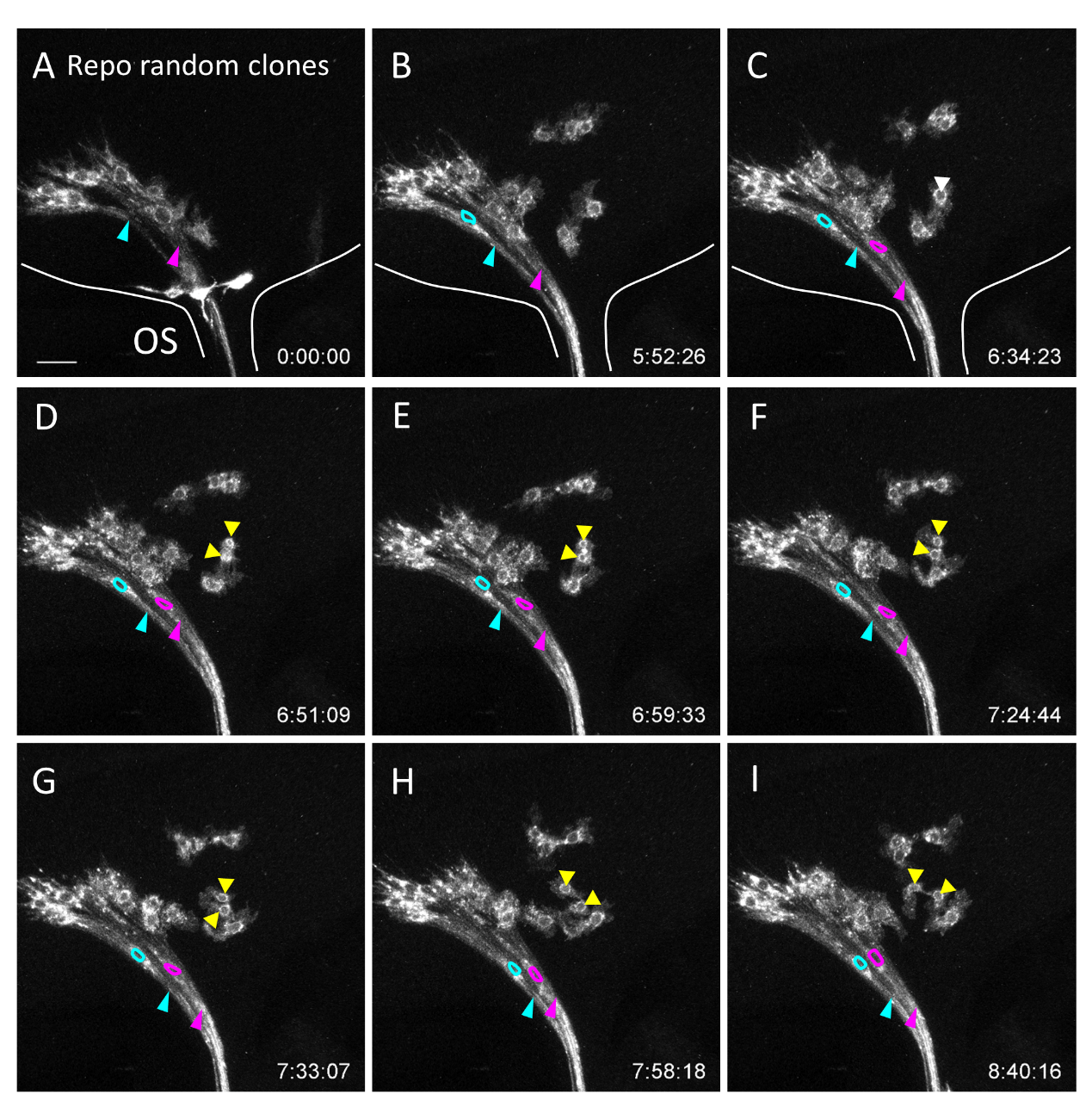


**Supplementary Figure 5. WG membrane extensions and nuclear movements.** Glia clones in eye disc were generated by hsFlp-induced flp-out and visualized by *repo-GAL4* driven *UAS-mGFP* (white) and includes both the elongated WG (cyan and magenta arrowheads) and the round SG (yellow arrowheads). The arrowheads indicate a young (left, cyan) and an old (right, magenta) WG, respectively. Images are shown with anterior to the top. (A) The younger WG (left) has short and thin membrane extension. The older WG membrane has reached the OS. (B-I) The younger WG membrane extended further toward the OS. The right WG membrane has thickened. The SG migrated more anteriorly. (C) The yellow arrowhead indicates a surface glia (SG) about to undergo mitosis. (D-H) The yellow arrowheads mark the two daughter cells derived from the dividing SG in (C). (A-I) The nucleus of the two WGs move up and down along the membrane extensions, but gradually toward the OS. Scale bar is 30μm for A-I.

**Supplementary Movie 2. Flp-out clones of wrapping glia and surface glia.** The clones were labeled by mCD8GFP driven by repo-GAL4. Eye disc is anterior to the top. Both WG and SG clones were found in this sample eye disc. Progressive membrane extension of WG was observed in the left-most one. Migratory SG was first found in the lower part of eye disc and moved up to the anterior portion of eye disc. Cell division can be found during SG migration. There is no anterior membrane extension of WG.


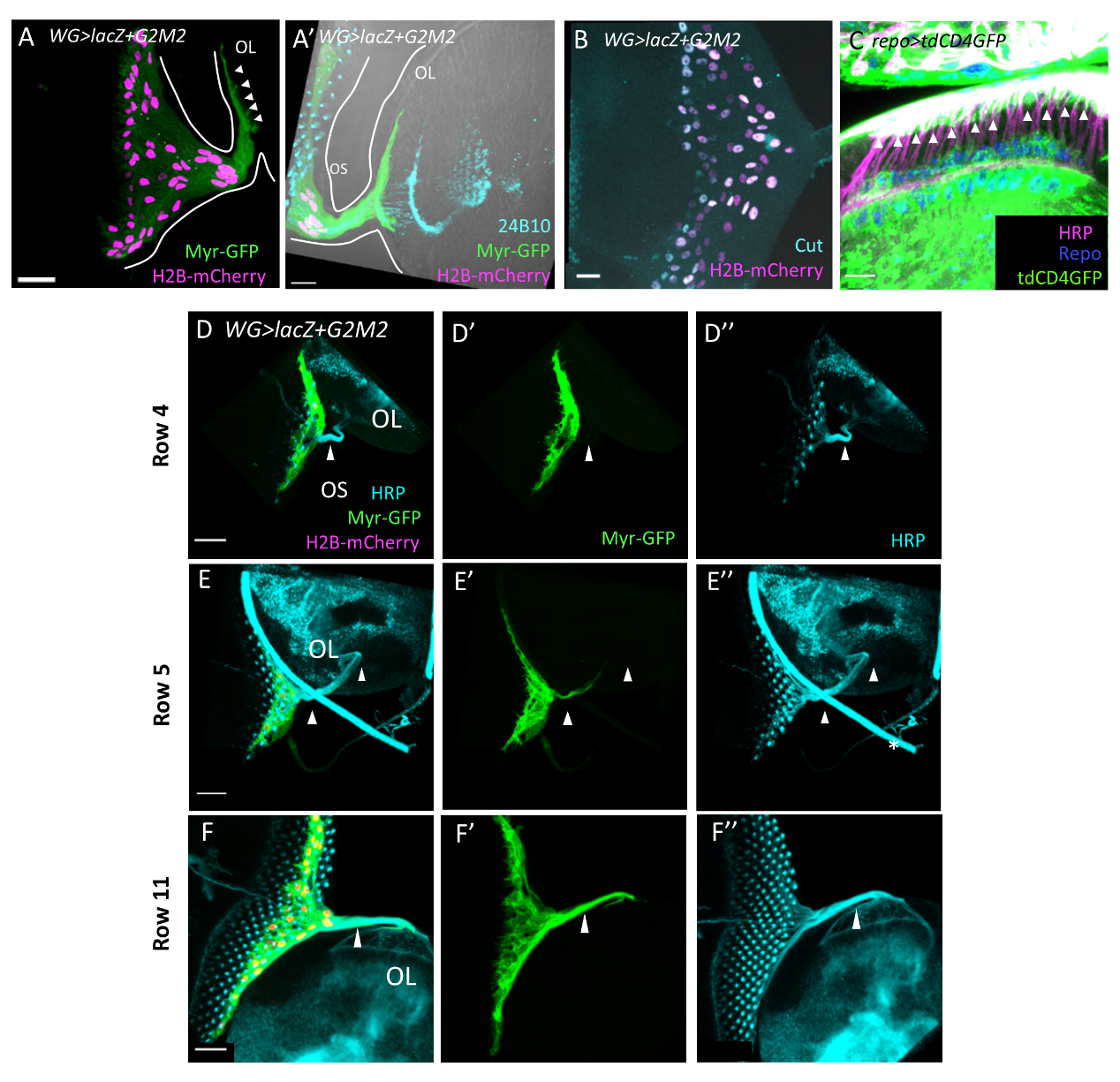


**Supplementary Figure 6. WG membrane extends after PR projection.** (A, B, D-F) *WG-GAL4* (*GMR74E02-GAL4*)*-*driven *UAS-lacZ-G2M2* expression, which contains myr‐GFP‐V5‐P2A and H2B‐mCherry‐HA, reveals WG membrane morphology (myr-GFP, green) and nucleus (H2B-mCherry, magenta). (A-A’) *WG-GAL4* is exclusively expressed in glia in the eye disc and not in the optic lobe. (B) Cut (cyan) staining co-localizes with nuclei labeled by *WG-GAL4,* demonstrating that *WG-GAL4* is only expressed in WG. (C) *tdCD4GFP* (green) driven by the pan-glia *repo-GAL4* demonstrates a membrane gap between WG (arrowheads) and lamina glia. HRP (magenta) stains axons. Repo (blue) stains glia nuclei. (D-F) Temporal progressions of PR axons projection (HRP, cyan) and glial membrane extension (myr-GFP, green). The age of eye disc is represented by the number of ommatidia rows. Arrowheads indicate the OS. (D-D”) In an eye disc with four rows of ommatidia, the PR axons have reached OS but not the lamina, and the WG membrane has not yet reached the OS. (E-E’’) In an eye disc with 5 rows of ommatidia, the PR axons have entered the lamina, and the WG membrane has entered OS but not the lamina. The asterisk indicates debris. (F-F’’) In an 11-row eye disc, The PR axons have spread out to occupy the lamina and formed the lamina plexus, and the WG membrane has reach the lamina. OS, optic stalk; OL, optic lobe. Scale bars are 20μm for A-C and 30μm for D-E.


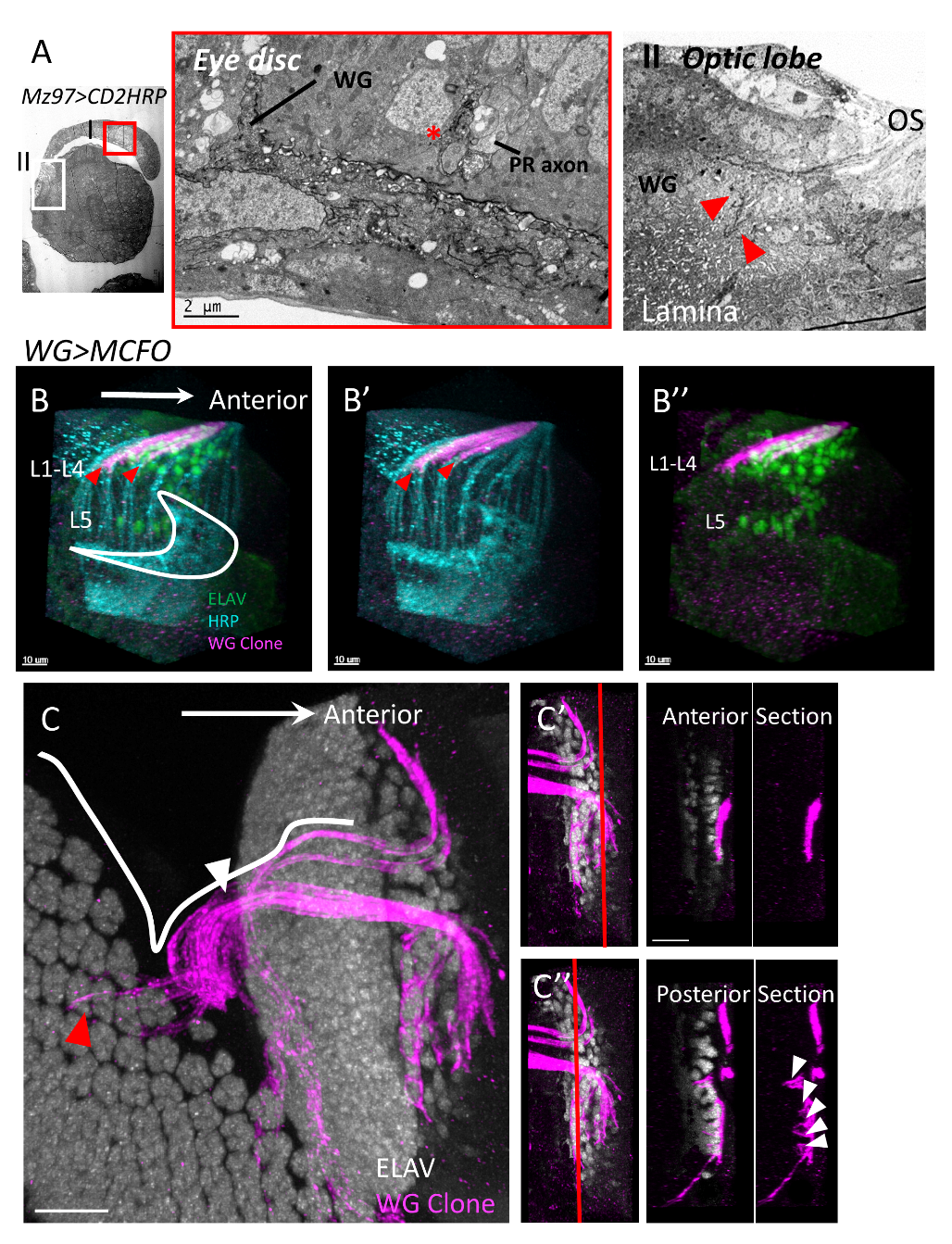


**Supplementary Figure 7. Wrapping Glia terminated at lamina neuron (L1-L4) layer and far above L5.** (A) Membrane bound CD2-HRP are driven by the WG-specific *Mz97-GAL4*. WG are outlined by DAB staining. In region I in eye disc, WG membrane is found penetrating to the PR layer. Next to the WG membrane to PR layer, a cross section of a single axon is wrapped by WG. Partial WG membrane between axonal bundles is indicated by red asterisk. In region II, WG membrane reaches to the lamina through optic stalk. (B-C’’) WG MCFO clones were generated by *WG-GAL4*. HRP (cyan) is used to show axon projection and position of lamina plexus (white curve). ELAV (green) stains for the nucleus of lamina neurons (L1-L4, L5). (B) The WG clones extend membrane to the L1-L4 but not reaching L5 and lamina plexus. (C) Anterior and posterior WG MCFO clones are indicated by red and white arrowhead respectively. ELAV stains for neuronal nuclei (white). (C’, C’’) Anterior and posterior optical sections (at the red line) are displayed respectively. (C’) In the anterior optical section, the WG membrane is adjacent to the lamina neurons. (C’’) In the posterior optical section, the WG membrane has extended to partially surround the lamina neurons (white arrowheads). Scale bars are 2μm for AI; 10μm for B-B’’; 20μm for C-C’’.


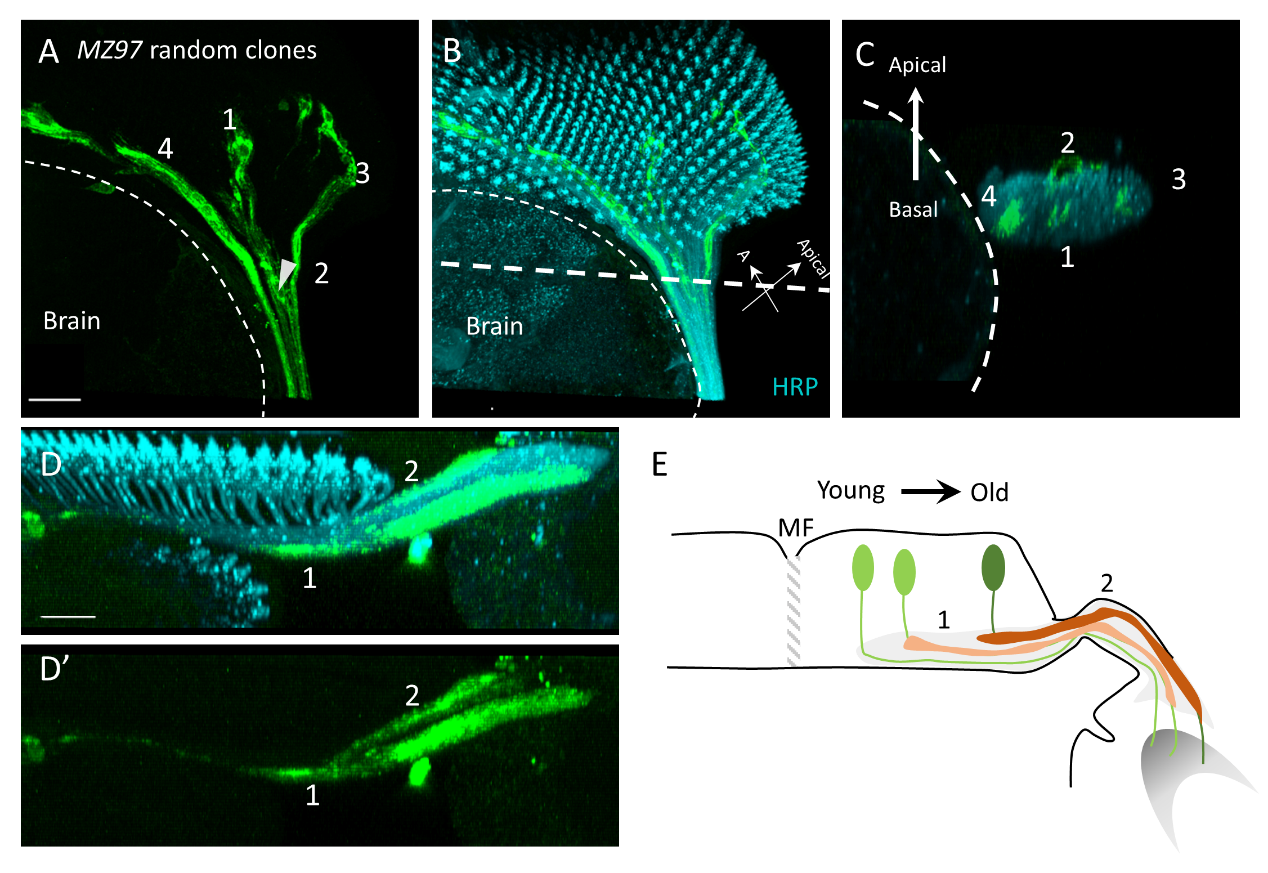


**Supplementary Figure 8. Young WG lay at more basal position in eye disc.** Flp-out WG clones were induced by *hs-Flp* and visualized by *Mz97-GAL4* driven mCD8-GFP (green)*.* HRP (cyan) shows PRs and PR axons. (A-B) The dashed line marks the outline of brain. Four single WG (#1-4) were labelled. (C) The optical cross section of the OS showed that the anterior WG (#1) membrane lay in the basal and the posterior WG (#2) membrane lay in the apical region. The two lateral WG (#3 and #4) maintained their relative D-V positions in the OS. (D, D’) The longitudinal cross section view of eye disc showed the membrane of WG #1 and #2. The anterior (younger, #1) membrane is more basal than the posterior (older, #2) membrane. (E) Schematic drawing shows the spatial correlation between axons of PRs (green/dark green) and membrane of WG (brown and dark brown) with different birth order. Scale bar is 30μm for AB; Scale bar is 10μm for CDD’.


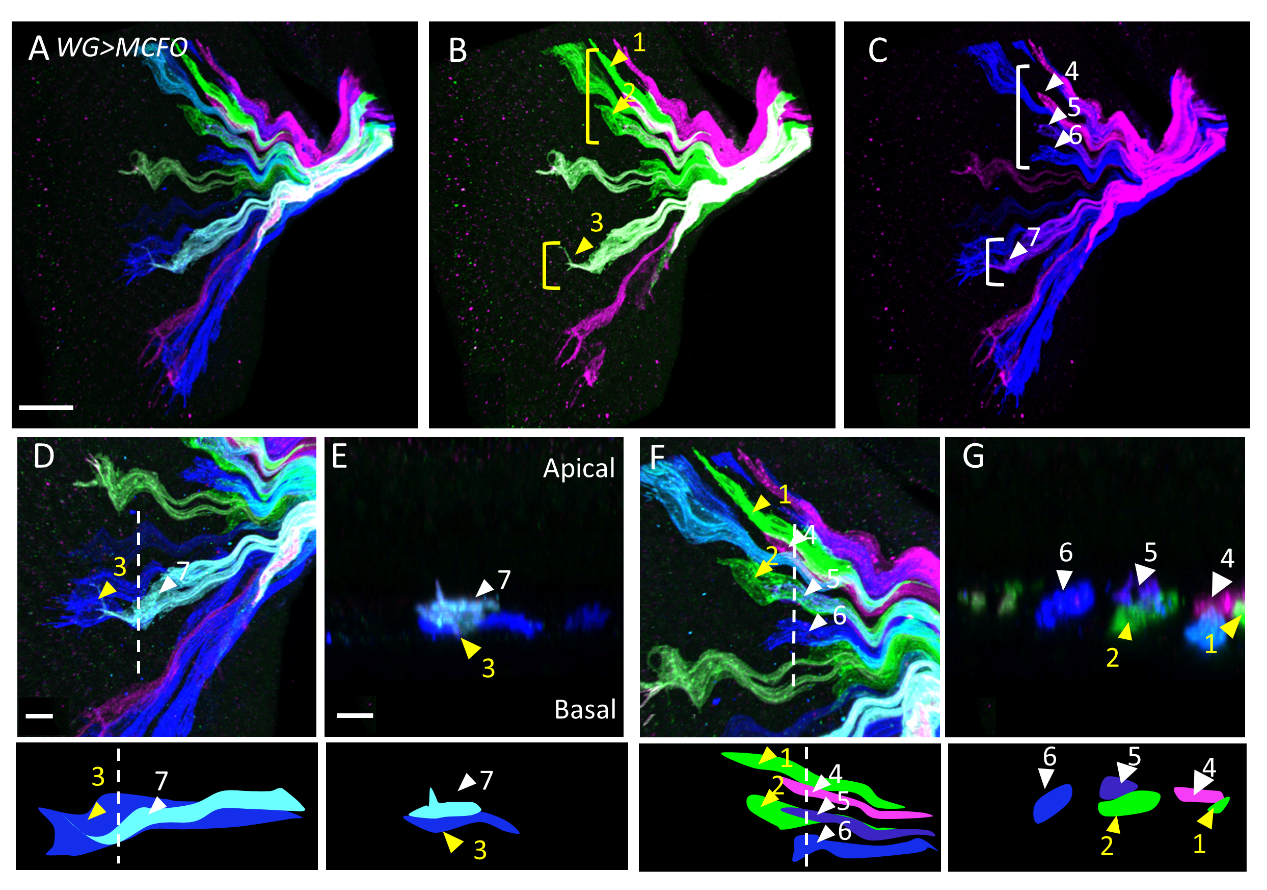


**Supplementary Figure 9. Retinotopic projection of WG membrane in OS and lamina.** (A-G) An eye disc with multiple WG MCFO clones. Anterior is to the right, and dorsal is to the top. Individual clones are numbered and shown in different panels. (B) The yellow brackets indicate anterior clones (#1-3). (C) The white brackets indicate more posterior clones (#4-7). (D, F) Higher magnification of the two regions shown in (B, C). (D) Clone 3 is more anterior to clone 7. (E) In lateral view along the white dashed line in (D), clone 7 is apical to clone 3. (F) Clones 1 and 2 are more anterior to clones 4-6. (G) In lateral view along the white dashed line in (F), clones 4-6 are more apical to clones 1 and 2. Scale bar are 30μm for A-C and 10μm for D-G.


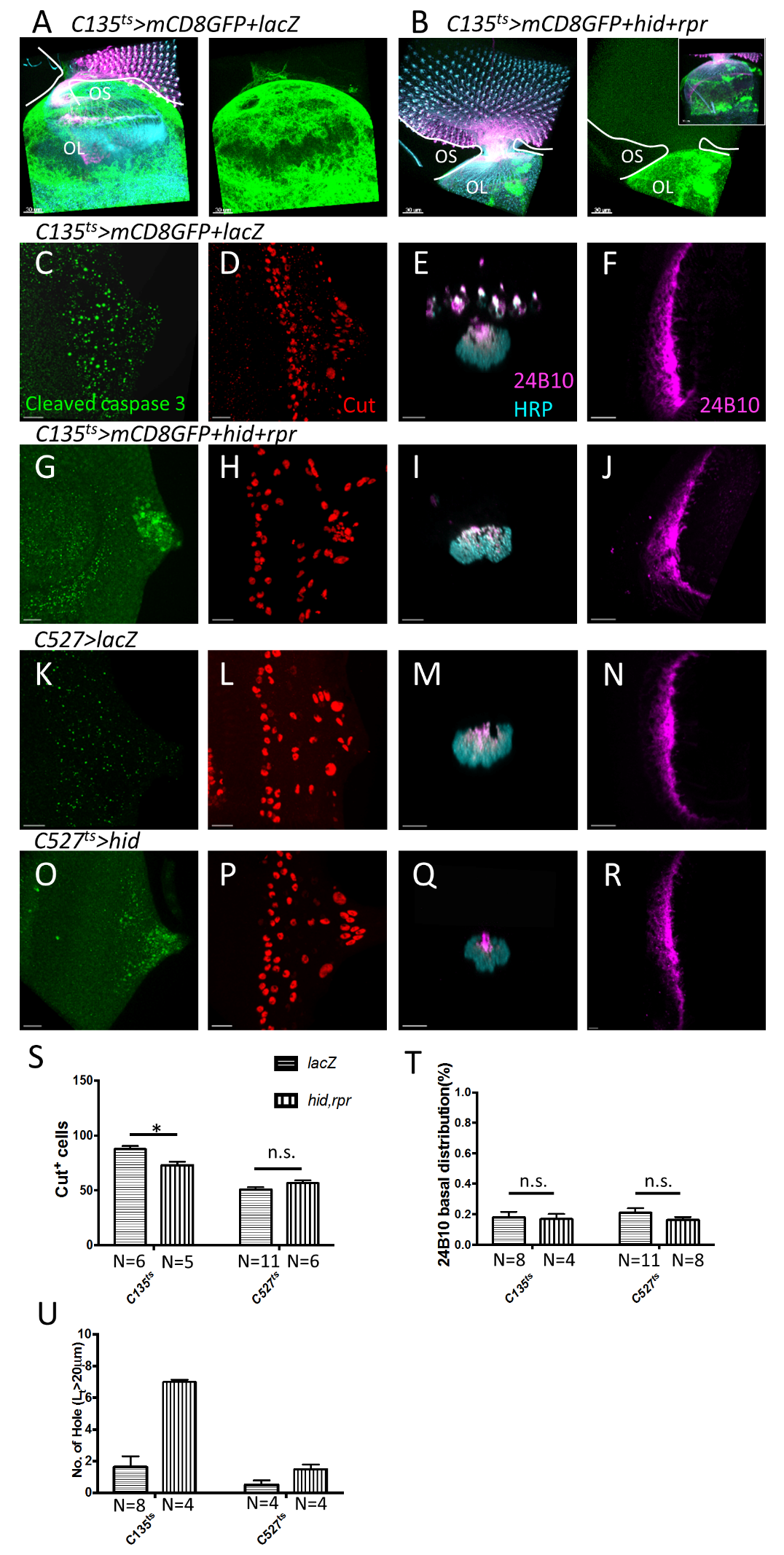


**Supplementary Figure 10.** **The effects of killing CG and SG on retinotopic projections of PR axons.** (A) The mCD8GFP (green) driven by the CG-specific *C135-GAL4* showed the extensive CG membrane in eye disc and optic lobe. (B) In *C135^ts^>mCD8GFP+hid+rpr*, the CG membrane (mCD8GFP, green) is absent in eye disc and largely reduced in optic lobe. The *C135^ts^>mCD8GFP+lacZ* (C-F) and *C527>lacZ* (K-N) serve as controls for CG and SG respectively. (G-J) The death genes *hid* and *rpr* were driven by the CG-specific *C135-GAL4*. (O-R) *hid* was driven by the SG-specific *C527-GAL4.* Since the continuous expression of death genes in *C135>hid+rpr* or *C527>hid* lead to larval death, we used *tub-GAL80^ts^* to temporally control the death genes expression. 12 hrs temperature shift to 30℃was used to transiently induce death genes expression. Cleaved caspase 3 (green), Cut (red) and 24B10/HRP (magenta/cyan) were detected. In contrast to *C135^ts^>mCD8GFP+lacZ* (C-F)*,* the *C135^ts^>mCD8GFP+hid+rpr* (G-J) showed strong cleaved caspase 3 signal (green in G) at the position of the two giant CG nuclei near optic stalk. (H) The number of WG (Cut^+^) is slightly reduced (quantitative analysis in S). (I) The apical distribution of young PR axons in the optic stalk is not disturbed (quantitative analysis in T). (J) The PR retinotopic projections in lamina plexus is disorganized, with larger holes in the lamina. In contrast to *C527>lacZ* (K-N)*,* the *C527^ts^>hid* (O-R) showed elevated cleaved caspase 3 signal (green). (P) The number of WG (Cut^+^) is not reduced (quantitative analysis in S). (Q) The apical distribution of young PR axons in the optic stalk is not disturbed (quantitative analysis in T). (R) The PR retinotopic projections in lamina plexus is more disorganized, with more larger holes in the lamina (quantitative analysis in U). Scale bars are 30μm for A-B; 20μm for C-R.


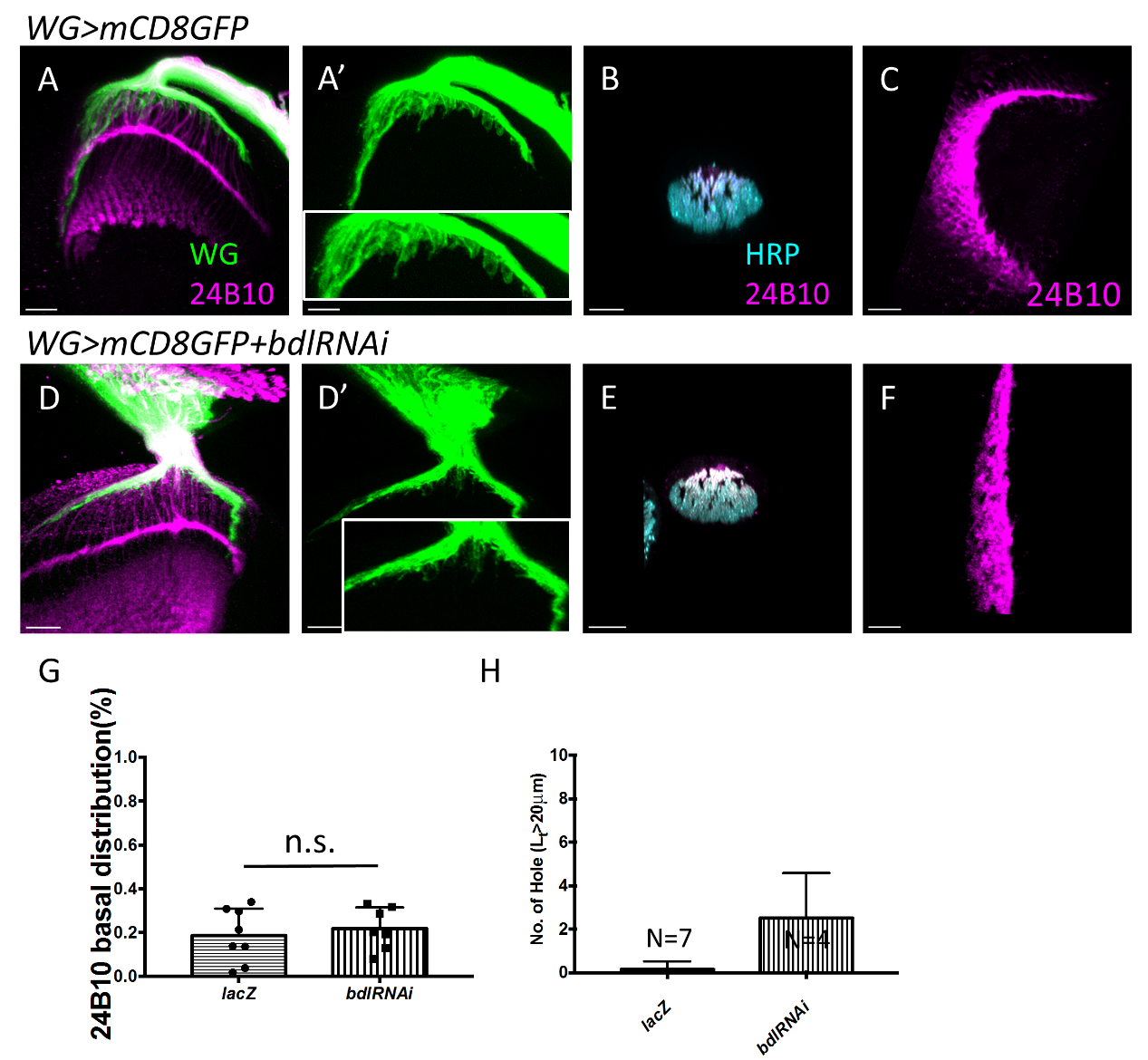


**Supplementary Figure 11. Knockdown of *Bdl* in WG caused aberrant retinotopic projection in lamina**. (A, D) *mCD8GFP* and *Bdl-RNAi* were driven by *WG-GAL4.* 24B10 (magenta) staining labels older axons. (A’, D’) As shown by previous study (Cameron et al., 2016), the level of WG membrane extension in lamina is reduced. (B, E) The distribution of 24B10/HRP (magenta/cyan) staining in OS is similar in *WG>mCD8GFP+Bdl-RNAi* and the control group (*WG>mCD8GFP*) (quantitative analysis in G). (C, F) The retinotopic projection pattern in lamina is more disorganized *WG>mCD8GFP+Bdl-RNAi* (quantitative analysis in H). Scale bars are 20μm for all.
